# TAp73-induced phosphofructokinase-1 transcription promotes the Warburg effect and enhances cell proliferation

**DOI:** 10.1038/s41467-018-07127-8

**Published:** 2018-11-08

**Authors:** Le Li, Lijia Li, Wei Li, Taiqi Chen, Bin Zou, Lina Zhao, Huili Wang, Xueying Wang, Lina Xu, Xiaohui Liu, Dong Wang, Bo Li, Tak W. Mak, Wenjing Du, Xiaolu Yang, Peng Jiang

**Affiliations:** 1 School of Life Sciences, Tsinghua University; Collaborative Innovation Center for Cancer Medicine, 100084 Beijing, China; 2 State Key Laboratory of Medical Molecular Biology, Institute of Basic Medical Sciences Chinese Academy of Medical Sciences, School of Basic Medicine, Peking Union Medical College, 100005 Beijing, China; 3 School of Medicine, Tsinghua University, 100084 Beijing, China; 4 Zhongshan School of Medicine, Sun Yat-sen University, 510630 Guangzhou, Guangdong China; 5 The Campbell Family Institute for Breast Cancer Research, Princess Margaret Hospital, Toronto, ON M5G 2C1 Canada; 6 Department of Cancer Biology and Abramson Family Cancer Research Institute, Perelman School of Medicine, University of Pennsylvania, Philadelphia, PA 19104 USA

## Abstract

The Warburg effect is a prominent metabolic feature associated with neoplastic diseases; however, the underlying mechanism remains incompletely understood. TAp73, a structural homolog of the tumor suppressor p53, is frequently overexpressed in human tumors, indicating a proliferative advantage that it can confer to tumor cells. Here we show that TAp73 stimulates the expression of phosphofructokinase-1, liver type (PFKL), which catalyzes the committed step in glycolysis. Through this regulation, TAp73 enhances glucose consumption and lactate excretion, promoting the Warburg effect. By activating PFKL, TAp73 also increases ATP production and bolsters anti-oxidant defense. TAp73 deficiency results in a pronounced reduction in tumorigenic potential, which can be rescued by forced PFKL expression. These findings establish TAp73 as a critical regulator of glycolysis and reveal a mechanism by which tumor cells achieve the Warburg effect to enable oncogenic growth.

## Abstract (web summary)

TAp73 is a structural homolog of the tumor suppressor p53. Here they show that TAp73 is critical for promoting glycolysis as it stimulates the transcriptional expression of liver type of phosphofructokinase-1 (PFKL), which catalyzes the committed step in glycolysis.

## Introduction

The uncontrolled and continuing proliferation characteristic of malignancies is intimately linked to the reprogramming of metabolic pathways, with the most notable feature being the Warburg effect or aerobic glycolysis^1–4^. Glycolysis converts glucose into pyruvate. In normal quiescent cells, pyruvate is oxidized to CO_2_ via mitochondrial oxidative phosphorylation, while it is processed to lactate only under anaerobic conditions, with a **~**18-fold lower efficiency of ATP production^5^. However, as Otto Warburg first reported in the 1920s, tumor cells consumed glucose at a markedly increased rate and excreted a large amount of lactate, even in the presence of sufficient oxygen^6,7^. The prevalence of the Warburg effect among tumor cells has been confirmed in the ensuing decades and exploited clinically with positron emission tomography (PET) for noninvasive imaging of a variety of solid tumors^8^. The Warburg effect is also observed in normal proliferating cells such as lymphocytes^9^. Accumulating evidence suggests that the Warburg effect is enabled by oncogenic mutations in tumor cells and by regulated growth factor signaling in normal cells, to facilitate biosynthesis and redox homeostasis required for cell growth and division^2–4,10^. However, both the causes of the Warburg effect and its coordination with the other major metabolic alterations in proliferating cells are not well understood.

The committed step in glycolysis is the phosphorylation of fructose 6-phosphate (F6P) to fructose 1,6-bisphosphate (F-1,6-BP) (Supplementary Fig. 1a). This reaction is catalyzed by phosphofructokinase-1 (PFK-1), which in humans exists in three isoforms: liver (L), muscle (M), and platelet (P)^5,11^. As the “pace-setter” of glycolysis, PFK-1 is the most important site of regulation^5,11^. PFK-1 activity is stimulated when the substrate F6P is abundant, due to PFK-2-mediated conversion of F6P to fructose 2,6-biphosphate (F-2,6-BP), a potent activator of PFK-1. In contrast, PFK-1 activity is inhibited by high levels of ATP and citrate, which signify sufficient energy charge and plentiful biosynthetic precursors, respectively. These allosteric regulators permit acute and temporary adjustment of glycolytic flux (Supplementary Fig. 1a). In addition, PFK-1 is regulated by post-translational modifications including glycosylation^12^, to achieve a more long-lasting, yet reversible, alteration. Moreover, PFK-1 is controlled at the level of expression to attain a persistent change in glycolytic flux. Especially, the expression of PFK-1 increases in proliferating cells, but declines upon withdrawal of growth factors^13^. In tumor cells, the expression of PFK-1 is often upregulated, and the composition of the isoforms changed, with PFKL and PFKP being more highly expressed compared PFKM^14^. Nevertheless, the mechanisms that control *PFK-1* expression in normal and malignant cells remain unknown.

p73 is a structurally homolog of p53, with cellular functions that both overlap and contrast with those of the preeminent tumor suppressor^15–18^. p73 is expressed in two major isoform classes (ΔN and TA) that are different in their N-terminal region due to the use of alternative promoters. ΔNp73 lacks an intact transactivation domain, while retaining the oligomerization and DNA-binding domains (Supplementary Fig. 1b). As such, ΔNp73 can act as a dominant negative inhibitor for the functionally active p53 family proteins by forming hetero-oligomers with them or by competing with them for binding to target genes. Hence, ΔNp73 is oncogenic^15,19^. In contrast, TAp73, like p53, contains an N-terminal transactivation domain and can activate p53-responsible genes. Deficiency in TAp73 leads to increased susceptibility to spontaneous and carcinogen-induced tumor formation, suggesting a tumor suppressive role of TAp73 (see ref. ^20^). Nevertheless, unlike p53 whose mutation is the single most frequent genetic lesion in human tumors, TAp73 is rarely mutated^15,17,18^. Instead, it is frequently upregulated, indicative of a proliferative advantage that TAp73 can afford to tumor cells. Consistently, TAp73 promotes mitochondrial respiration^21^, serine biosynthesis^22^, and angiogenesis^23^. We previously showed that TAp73 regulates the pentose phosphate pathway (PPP), which branches off glycolysis at glucose-6-phosphate (Supplementary Fig. 1a)^5,24^. TAp73 activates the expression of glucose-6-phosphate dehydrogenase (*G6PD*), which encodes the rate-limiting enzyme of the PPP^25^. Nevertheless, overexpression of G6PD does not completely rescue the defects of TAp73-deficient cells^25^, implying the involvement of an additional TAp73 target(s) in cell proliferation.

Here we investigate the role of TAp73 in glucose metabolism and identify a critical role for TAp73 in the activation of the liver isozyme of PFK-1 (PFKL). By regulating PFKL, TAp73 enhances glucose consumption and lactate excretion. Ectopic expression of PFKL, like G6PD, can restore the tumorigenic potential of TAp73-deficient cells. TAp73 is activated in response to mitogens, thereby coupling growth factor signaling with glycolysis. Moreover, upregulation of TAp73 correlates with higher PFKL expression in tumor cells. These findings establish TAp73 as a critical regulator of glucose metabolism, promoting Warburg effect and coordinating glycolysis with the PPP to enable cell proliferation.

## Results

### TAp73 enhances glycolysis and promotes the Warburg effect

**Fig. 1:**
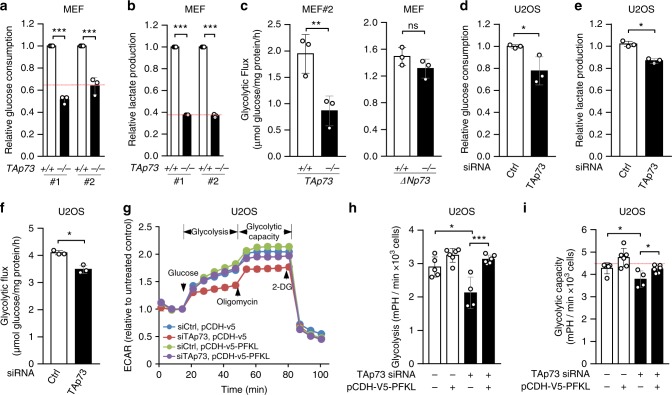
TAp73 promotes the Warburg effect. **a**, **b** Glucose consumption (**a**) and lactate excretion (**b**) in two independent pairs (#1 and #2) of *TAp73*^*+/+*^ and *TAp73*^*−/−*^ MEFs. Data are means ± S.D. (*n* = 3 independent experiments). **c** *TAp73*^*+/+*^ and *TAp73*^*−/−*^ MEFs (left), or *ΔNp73*^*+/+*^ and *ΔNp73*^*−/−*^ MEFs (right) were cultured in medium containing [1,2–^13^C_2_]glucose. Glycolytic flux was measured on the base of glucose consumption rate and the generation of ^13^C-labeling lactate (M2) determined by LC-MS. Data are means ± S.D. (*n* = 3). **d**–**f** U2OS cells treated with control (Ctrl) or *TAp73* siRNA were assayed for glucose consumption (**d**), lactate excretion (**e**), and glycolytic flux (**f**). Data are means ± S.D. (*n* = 3) **g**–**i**, U2OS cells stably transfected with control plasmid (pCDH-V5) or PFKL plasmid (pCDH-v5-PFKL) were treated with control or TAp73 siRNA. Cells were supplied with 10 mM glucose, 2 μM oligomycin, and 100 mM 2-DG at the indicated times. ECAR was examined using Seahorse XFe96 analyzer (**g**). Relative glycolysis levels (**h**) and glycolytic capacity (**i**) are normalized to the cell number (means ± S.D., *n* = 4-6)

We previously found that TAp73 enhances the PPP by stimulating the expression of G6PD^25^. As this regulation did not fully explain the effect of TAp73 on cell proliferation^25^, we investigated whether TAp73 also regulates glycolysis. Using two independent pairs of E1A/H-Ras^V12^-transformed mouse embryonic fibroblasts (MEFs) (Supplementary Fig. 2a)^20^, we observed that cells with homozygous deletion of *TAp73* (*TAp73*^*−/−*^) displayed ~40–60% reduction in glucose consumption (Fig. 1a) and lactate excretion (Fig. 1b) compared to the corresponding wild type (*TAp73*^*+/+*^) cells. To evaluate the effect of TAp73 on glycolytic flux, we cultured *TAp73*^*+/+*^ and *TAp73*^*−/−*^ MEFs in medium containing [1,2–^13^C_2_]glucose and measured incorporation of ^13^C in lactate using liquid chromatography-mass spectrometry (LC-MS). Deficiency in *TAp73* reduced glycolytic flux by ~60% (Fig. 1c, left). The effect on glycolysis was specific to the TA isoform, as E1A/Ras^V12^-transformed *ΔNp73*^*+/+*^ and *ΔNp73*^*−/−*^ MEFs^26^ showed no significant difference in glycolytic flux (Fig. 1c, right).

To determine the effect of TAp73 on glycolysis in human tumor cells, we knocked down TAp73 in U2OS osteosarcoma cells using small interfering RNA (siRNA) (Supplementary Fig. 2b). This led to a significant reduction in glucose consumption (Fig. 1d), lactate excretion (Fig. 1e), and glycolytic flux (Fig. 1f). To corroborate these results, we evaluated glycolysis and glycolysis capacity by measuring extracellular acidification rate (ECAR), under conditions where cells were supplied sequentially with glucose to feed glycolysis, the ATP synthase inhibitor oligomycin to drive glycolysis to the maximal capacity, and the glucose analog 2-deoxyglucose (2-DG) to block glycolysis (Fig. 1g). U2OS cells devoid of TAp73 displayed substantially reduced glycolysis and glycolysis capacity compared to control cells (Fig. 1g–i). Similarly, knocking down TAp73 in several other human cell lines including A172 and SF188 glioma cells, as well as H1299 and A549 lung cancer cells, resulted in a noticeable reduction in glucose consumption and lactate excretion (Supplementary Fig. 2c–k). These results indicate that TAp73 enhances glycolysis and promotes the Warburg effect.

### TAp73 activates the expression of PFKL

**Fig. 2:**
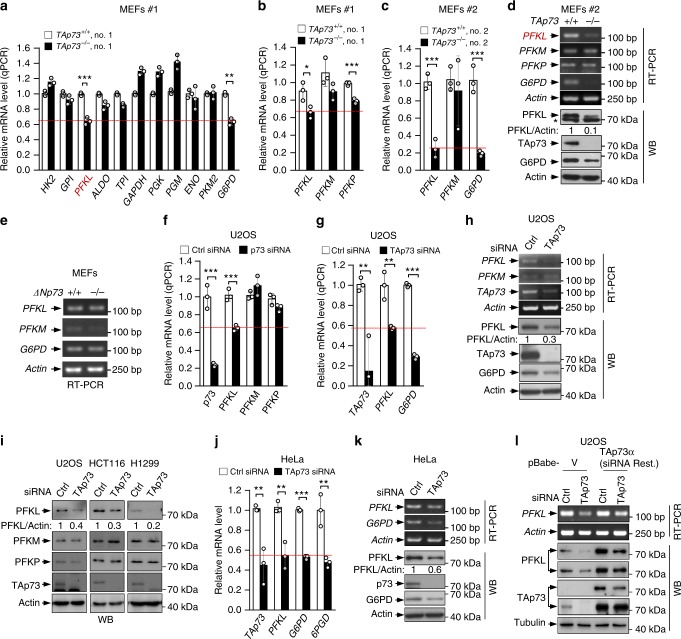
TAp73 regulates the expression of PFKL. **a** Relative mRNA levels of glycolytic enzymes in *TAp73*^*+/+*^ and *TAp73*^*−/−*^ MEFs (clone #1), as analyzed by qRT-PCR. Data are means ± S.D. (*n* = 3). **b**, **c** *TAp73*^*+/+*^ and *TAp73*^*−/−*^ MEFs (clone #1 and #2) were analyzed by qRT-PCR for expressions of PFKL, PFKM, and PFKP, and G6PD. Data are means ± S.D. (n = 3). **d** *TAp73*^*+/+*^ and *TAp73*^*−/−*^ MEFs (clone #2) were analyzed by semi-quantitative RT-PCR (top), and Western blot (WB, bottom). **e** mRNA levels of PFKL, PFKM, and G6PD in *ΔNp73*^+/+^ and *ΔNp73*^−/−^ MEF cells. Results are representative of three independent experiments. **f** U2OS cells were transfected with control or p73 siRNA as indicated. mRNA expression was detected by qRT-PCR (means ± S.D., *n* = 3). **g**, **h** U2OS cells were transfected with control or TAp73 siRNA were analyzed by qRT-PCR (**g**, means ± S.D., *n* = 3), semi-quantitative RT-PCR (**h**, top), and Western blot (WB) (**h**, bottom). **i** U2OS, HCT116, and H1299 cells transfected with control or TAp73 siRNA were analyzed by Western blot. Results are representative of three independent experiments. **j**, **k** HeLa cells transfected with control or TAp73 siRNA were analyzed for mRNA and protein expression. Data are means ± S.D. (*n* = 3). **l** U2OS cells stably expressing control plasmid or an siRNA-resistant TAp73 plasmid were transfected with a control or TAp73 siRNA. PFKL expression was analyzed by RT-PCR and Western blotting

To investigate the mechanism by which TAp73 regulates glycolysis, we compared the expression of several major glycolytic enzymes in *TAp73*^*−/−*^ versus *TAp73*^*+/+*^ MEFs. The mRNA levels of most enzymes either remained approximately the same (HK2, GPI, ALDO, TPI, ENO, and PKM2) or increased moderately (GAPDH, PGK, and PGM) in *TAp73*^*−/−*^ MEFs (Fig. 2a). Interestingly, however, the mRNA levels of PFKL were substantially decreased, as shown by both quantitative reverse transcription polymerase chain reaction (qRT-PCR) (Fig. 2a–c) and semi-quantitative RT-PCR (Fig. 2d, top). Accordingly, PFKL protein levels were found to be much lower in *TAp73*^*−/−*^ than in *TAp73*^*+/+*^ MEFs (Fig. 2d, bottom). In contrast, *ΔNp73*^*+/+*^ and *ΔNp73*^*−/−*^ MEFs had comparable levels of PFKL (Fig. 2e).

Similarly, knocking down TAp73 in U2OS cells resulted in a strong decrease in both mRNA and protein levels of PFKL (Fig. 2f–i). To verify the generality the effect of TAp73 in human cells, we knocked down TAp73 in H1299 cells and observed a strong reduction in PFKL expression (Fig. 2i and Supplementary Fig. 3a, b). Moreover, upon TAp73 knockdown, PFKL protein and/or mRNA levels declined in cervical cancer HeLa cells (Fig. 2j, k) and colon cancer HCT116 cells (Fig. 2i and Supplementary Fig. 3c). Expression of an siRNA-resistant form of TAp73 not only restored PFKL levels in TAp73-knockdown cells (Fig. 2l, lanes 4 vs. 2) underscoring the specificity of the siRNA, but also markedly increased the levels of PFKL in control cells (Fig. 2l, lanes 3 vs. 1) confirming the stimulatory effect of TAp73 on PFKL.

In addition to PFKL, PFK-1 exists in two other isoforms, PFKM (muscle) and PFKP (platelet)^11^. However, deletion or knockdown of TAp73 showed minimal or no effect on the expression of PFKM and PFKP in MEFs (Fig. 2b–d) as well as various human cancer cell lines (Fig. 2f, h, i). Thus, the stimulatory effect of TAp73 appears to be specific for the PFKL isoform.

In MEFs and various human cell lines deficient in TAp73, PFKL was reduced to an extent comparable to G6PD (Fig. 2a, c, d, g, h, and Supplementary Fig. 3b), further supporting the notion that PFKL is a physiologically relevant target of TAp73. Of note, TAp73 deficiency also reduced the expression of 6-phosphogluconate dehydrogenase (6PGD) (Fig. 2j and Supplementary Fig. 3a), the second NADPH-generating enzyme in the PPP (Supplementary Fig. 1a)^5^, indicating coordinated regulation the two key enzymatic steps of the PPP by TAp73.

### TAp73 regulates PFKL under stressed conditions

**Fig. 3:**
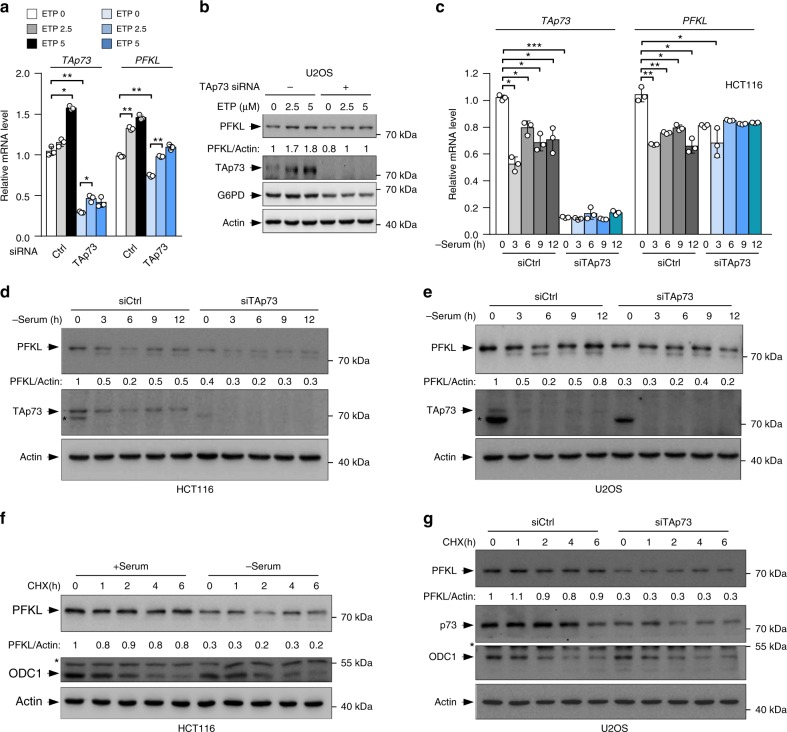
TAp73 regulates *PFKL* under stressed conditions. **a**, **b** U2OS cells transfected with control or TAp73 siRNA were treated with increasing amounts of etoposide (ETP) for 24 h, and analyzed by qRT-PCR (**a**, means ± S.D., *n* = 3) and Western blot (**b**). Data are representative of three independent experiments. **c**–**e** HCT116 (**c**, **d**) and U2OS (**e**) cells transfected with control siRNA or TAp73 siRNA were cultured in complete medium for 24 h and then in serum-free medium for different times. Cells were analyzed by qRT-PCR (**c**, means ± S.D., *n* = 3) and Western blot (**d**, **e**). **f** HCT116 cells were cultured in medium containing serum or no serum for 3 h, and then subjected to cycloheximide (CHX) chase in the presence or absence of serum. Whole cell extracts were collected using a pellet buffer as described previously^32^. Data are representative of three independent experiments. **g** U2OS cells transfected with control siRNA or TAp73 siRNA were subjected to CHX chase. Data are representative of three independent experiments

The expression of both TAp73 and PFKL is dynamically regulated. Especially, TAp73 is upregulated upon DNA damage^27–30^, while PFKL is downregulated in response to serum withdrawal^13^. When U2OS cells were treated with the genotoxic agent etoposide (ETP), levels of TAp73 mRNA and protein increased in an ETP concentration-dependent manner (Fig. 3a, b). Of note, the expression of PFKL increased in parallel in a TAp73-dependent manner (Fig. 3a, b). Moreover, when HCT116 cells were cultured in serum-deprived medium, levels of *PFKL* mRNA and protein declined rapidly initially (0–6 h) and partially recovered later (~9–12 h) (Fig. 3c, d). These changes were also highly correlative with the levels of TAp73 mRNA and protein, and depletion of TAp73 not only reduced the basal levels of PFKL, but also largely eliminated the fluctuation in PFKL expression (Fig. 3c, d). Similarly, levels of PFKL changed in U2OS cells during serum withdrawal in a TAp73-dependent manner (Fig. 3e). Consistent with TAp73-mediated transcriptional regulation of PFKL, the stability of the PFKL protein was not affected by serum deprivation or TAp73 depletion, as shown by cycloheximide (CHX) chase assays (Fig. 3f, g). Together, these results indicate that TAp73 controls *PFKL* expression in response to DNA damage and growth factor withdrawal.

### *PFKL* is a target gene for TAp73

**Fig. 4:**
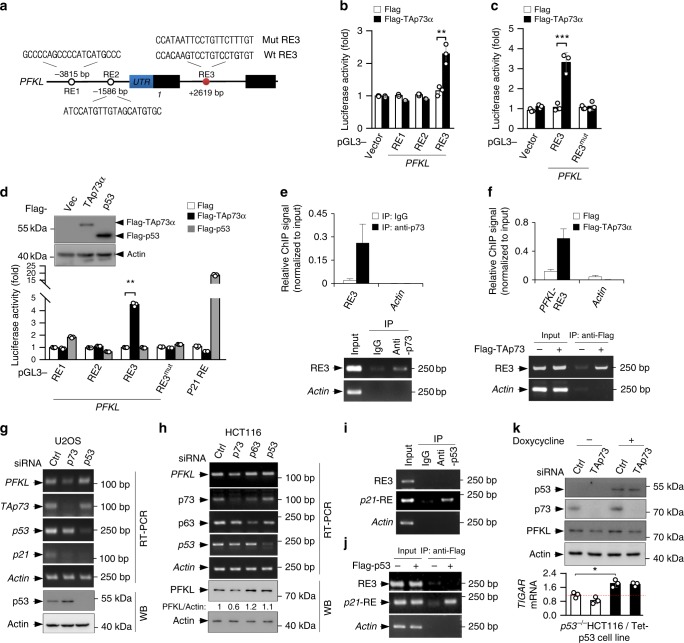
*PFKL* is a physiologically relevant target of TAp73. **a** Schematic representation of human *PFKL* genomic structure. The sequences of potential p73 response elements RE1–3 and the corresponding mutant RE3 are shown. **b**, **c** Luciferase constructs containing RE1, RE2, and RE3 (**b**), or RE3 and mutant RE3 (**c**) were transfected into 293T cells together with Flag-TAp73α or vector control. Renilla vector pRL-CMV was used as a transfection internal control. The relative luciferase activity was normalized to the co-transfected *Renila* activity. Data are means ± S.D. (*n* = 3). **d** Luciferase reporter constructs containing RE1, RE2, RE3, or RE3mut were transfected into 293T cells together vector control, p53, or TAp73. Renilla vector pRL-CMV was used as a transfection internal control. Relative levels of luciferase are shown. Data are means ± S.D. (*n* = 3). Insert shows protein expression. **e**, **f** U2OS cells (**e**), or 293T cells transfected with control vector or Flag-TAp73 (**f**), were analyzed by ChIP assay using normal mouse IgG and anti-p73 antibody (**e**), or anti-Flag antibody (**f**). Bound DNA was amplified by PCR and quantified. Results are representative of three independent experiments. **g**, **h** U2OS (**g**) and HCT116 (**h**) cells transfected with the indicated siRNAs were analyzed for protein and mRNA expression. Results are representative of three independent experiments. **i**, **j** U2OS cells (**i**), or 293T cells transfected with Flag-p53 or vector control (**j**), were analyzed by ChIP assay using normal mouse IgG and anti-p53 antibody (**i**), or anti-Flag antibody (**j**). Bound DNA was amplified by PCR and quantified. Results are representative of three independent experiments. **k** *p53*^*−/−*^ HCT116 cells stable expressing Tet-inducible p53 were cultured in medium containing [1,2–^13^C_2_]glucose and treated with doxycycline to induce p53 expression (Tet-on). p53, p73 and PFKL expressions were determined by Western blot analysis. TIGAR expression was analyzed by qRT-PCR. (means ± S.D., *n* = 3). Relative glycolytic flux is shown in Supplementary Fig. 4b

To evaluate whether TAp73 is a transcriptional activator for *PFKL*, we analyzed human *PFKL* gene sequence for potential response elements (REs) of p53 family proteins, which share the consensus sequence of 5′-RRRCWWGYYY-(0–13 base pair spacer)-RRRCWWGYYY-3′ (where R is a purine, Y a pyrimidine, and W an A or T)^32^. We identified two potential response elements (RE1 and RE2) in the 5′ flanking region, and one (RE3) in the first intron (Fig. 4a). We cloned the genomic fragment encompassing each response element into the promoter region of a firefly luciferase reporter plasmid, and found that TAp73 induced luciferase expression driven by RE3, but not RE1 or RE2 (Fig. 4b). Furthermore, TAp73 failed to induce luciferase expression driven by a mutant RE3 (RE3^mut^) (Fig. 4c, d), in which four conserved nucleotides were altered (Fig. 4a). Chromatin immunoprecipitation (ChIP) assays showed that both endogenous p73 (Fig. 4e) and Flag-TAp73α (Fig. 4f) associated with the RE3 region of *PFKL* in cells. These results indicate that TAp73 stimulates *PFKL* expression via binding to RE3.

Despite the structural similarity between TAp73 and p53, silencing p53 did not alter PFKL expression (Fig. 4g, h, and Supplementary Fig. 4a). Moreover, p53 was unable to stimulate luciferase expression driven by *PFKL* RE1, RE2, or RE3 (Fig. 4d). Also, neither endogenous and exogenous p53 could occupy the RE3 region of *PFKL* (Fig. 4i, j). To examine whether p53 affects TAp73-mediated PFKL expression, we used a *p53*^*−/−*^ HCT116 cell line containing an inducible (Tet-On) p53. Knockdown of TAp73 resulted in a decrease in both PFKL abundance (Fig. 4k) and glycolytic flux (Supplementary Fig. 4b), with or without p53 induction. Moreover, silencing the other p53 family member, p63, did not affect PFKL expression (Fig. 4h). Collectively, these results show that TAp73, but not p53 or p63, regulates the expression of the *PFKL* gene.

### TAp73 enhances glycolysis via PFKL

**Fig. 5:**
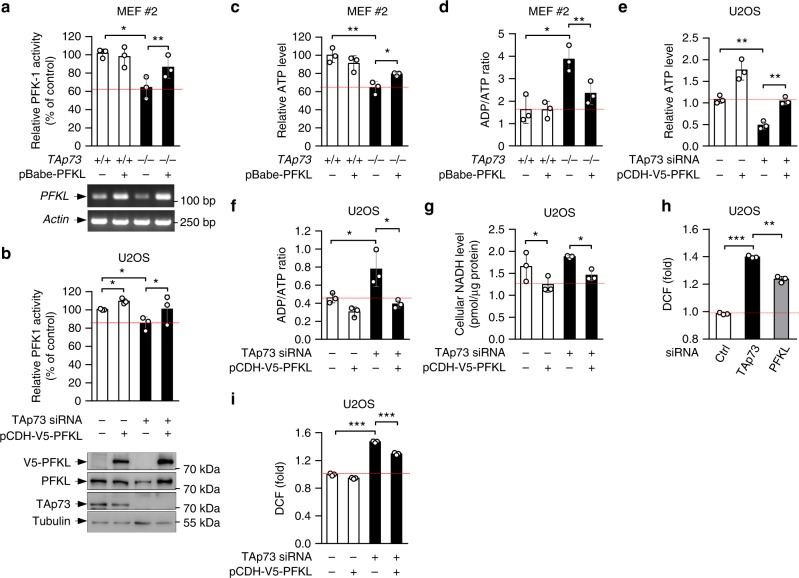
TAp73 regulates glycolysis, ATP production, and ROS homeostasis. **a**, **c**, **d** PFK-1 activity (top; means ± S.D., *n* = 3) and PFKL mRNA levels (bottom) (**a**), ATP levels (**c**), and ADP/ATP ratio (**d**) in *TAp73*^*+/+*^ and *TAp73*^*−/−*^ MEF cells stably expressing control vector or PFKL. **b**, **e**–**g**, **i** U2OS cells stably expressing control vector or PFKL were transfected with control or TAp73 siRNA. Shown are PFK-1 activity (top; means ± S.D., *n* = 3) and PFKL protein levels (bottom) (**b**), relative ATP levels (**e**), ADP/ATP ratio (**f**), NADH levels (**g**), and relative ROS content (**i**). Data are means ± S.D. (n = 3) **h** U2OS cells treated with control, TAp73, or PFKL siRNA were assayed for ROS accumulation by 2′7′-dichlorodihydrofluorescein diacetate (DCF) staining and FACS analysis. Data are means ± S.D., *n* = 3

PFK-1 catalyzes the irreversible conversion of fructose 6-phosphate to fructose 1,6-bisphosphate, committing glucose to glycolysis (Supplementary Fig. 1a). Deletion or knockdown of TAp73 significantly reduced overall PFK-1 activity in MEF, U2OS, and H1299 cells (Fig. 5a, b, and Supplementary Fig. 5a–c). The specificity of the assay was shown by an increase in the detected activity upon forced PFKL expression (Supplementary Fig. 5d). Forced expression of PFKL also largely restored PFK-1 activity in *TAp73*^*−/−*^ MEFs (Fig. 5a), as well as PFK-1 activity (Fig. 5b), glycolysis, and glycolytic capacity (Fig. 1g, h) in TAp73-knockdown U2OS cells. Consistent with its role in glycolysis, PFKL was detected along with PFKM and PFKP in several cell lines including MEF, U2OS, HeLa, H1299, and HCT116 (Supplementary Fig. 6a). Silencing PFKL reduced glucose consumption and lactate excretion. Silencing PFKM and PFKP achieved a similar effect, although in a cell type-dependent manner (Supplementary Fig. 6c–g). Collectively, these results show that TAp73 promotes glycolysis by upregulating *PFKL*.

Glycolysis is a main source of ATP in tumor cells. The lack of *TAp73* in MEFs resulted in a strong reduction in ATP levels, with a concomitant increase in the ADP/ATP ratio (Fig. 5c, d). A similar result was observed when TAp73 was depleted in U2OS cells (Fig. 5e, f). Ectopic expression of PFKL in *TAp73*^*−/−*^ MEFs and TAp73-depleted U2OS cells restored ATP levels (Fig. 5c, e) and reduced the ADP/ATP ratio (Fig. 5d, f), despite that its effect on the corresponding TAp73-proficient cells varied. Therefore, by activating PFKL and glycolysis, TAp73 enhances ATP production.

Previous studies showed that TAp73 can reduce NADH in part by activating oxidative phosphorylation^21^. Indeed, NADH levels were increased in TAp73-knockdown U2OS cells (Fig. 5g). Of note, forced expression of PFKL not only reduced NADH in control cells but also largely prevented NADH increase in TAp73-knockdown cells (Fig. 5g). Therefore, TAp73 likely reduces cellular NADH levels in part by stimulating PFKL and anaerobic glycolysis. TAp73 enhances NADPH production via the activation of the PPP^25^. Consistently, knockdown of TAp73 decreased NADPH (Supplementary Fig. 7a). However, forced expression of PFKL did not significantly alter NADPH levels in either control or TAp73-knockdown cells (Supplementary Fig. 7a). Thus, TAp73 elicits opposing effects on NADH and NADPH levels through the activation of glycolysis and the PPP, respectively.

TAp73 counteracts oxidative stresses in part through the activation of G6PD and the mitochondrial complex IV subunit Cox4i1 (cytochrome *c* oxidase subunit 4)^21,25^. As expected, depletion of TAp73 in U2OS cells elevated cellular ROS, as measured by the radical dye dichlorofluorescein diacetate (DCF) (Fig. 5h, i, and Supplementary Fig. 7b, c). Depletion of PFKL also increased ROS (Fig. 5h and Supplementary Fig. 7b). Conversely, forced expression of PFKL, while not affecting ROS in control cells, significantly reversed the rise of ROS in TAp73-depleted cells (Fig. 5i and Supplementary Fig. 7c). Thus, the effect of TAp73 on cellular ROS homeostasis is also mediated in part by PFKL. Collectively, these results show that TAp73 increases glycolysis through the activation of PFKL, thereby enhancing ATP production, reducing NADH levels, and ameliorating oxidative stress.

### TAp73 supports tumor cell proliferation by activating PFKL

**Fig. 6:**
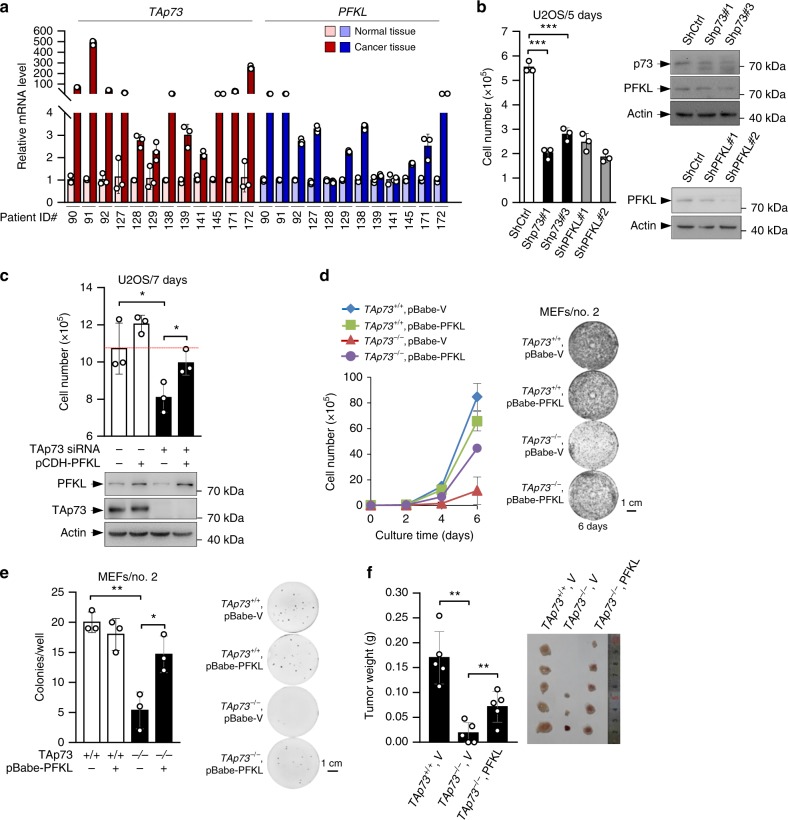
TAp73 promotes tumorigenesis through PFKL. **a** Expressions of TAp73 and PFKL in human colon tumors (C) and normal colon tissues (N) were analyzed by qRT-PCR. Patient ID numbers are shown. **b** U2OS cells stably expressing control or each of the two independent shRNAs for p73 or PFKL shRNA were analyzed for proliferation (left) and protein expression (right, means ± S.D., *n* = 3). **c** U2OS cells stably expressing control vector or PFKL were treated with or without TAp73 siRNA. Proliferation (top), and protein expression (bottom) were assayed. Experiments were repeated at least three times. **d** Proliferation of *TAp73*^*+/+*^ and *TAp73*^*−/−*^ MEF cells stably expressing PFKL or vector control (left, means ± S.D., *n* = 3), and representative images of cells stained with crystal violet at day 6 (right). **e** Left: Colony formation assay of *TAp73*^*+/+*^ and *TAp73*^*−/−*^ MEF cells stably expressing PFKL or vector control. Numbers of colonies with a diameter greater than 20 μm were quantified (means ± S.D., *n* = 3). Right: Representative images of colonies stained with crystal violet at day 6. **f** Average weights (left) and images (right) of xenograft tumors (3 weeks, means ± S.D., *n* = 5) generated by *TAp73*^*+/+*^ and *TAp73*^*−/−*^ MEF cells stably expressing PFKL or vector control as indicated

PFK-1 activity is markedly increased in some tumor cell lines^14^. A survey of public gene-expression databases (http://www.oncomine.org) revealed that the expression of PFKL was also significantly upregulated in several human cancers (Supplementary Fig. 8a). Moreover, elevated expression of PFKL or TAp73 significantly correlated with poor prognosis of lung cancer patients (Supplementary Fig. 8b, c). To extend these analyses, we examined 12 pairs of matched human normal and malignant colon samples. Interestingly, TAp73 expression was highly elevated in eight of the tumor samples compared to the normal counterparts (Fig. 6a). In almost all eight cases, the expression of PFKL was concomitantly increased (Fig. 6a), while the expression of both TAp73 and PFKL was low in the remaining cases, indicating up-regulation of and positive correlation between TAp73 and PFKL expression in colon cancer.

Homozygous deletion of *TAp73* in the E1A/H-Ras^V12^-transformed MEFs markedly slowed down adherent proliferation on plate (Supplementary Fig. 9a)^25^ and anchorage-independent growth in soft agar (Supplementary Fig. 9b, c)^25^, an in vitro measure of tumorigenicity. A similar effect was observed when TAp73 was stably knocked down in U2OS cells by shRNA (Fig. 6b)^25^. Moreover, TAp73 knockdown strongly reduced proliferation of A172, SF188, H1299, and A549 cells (Supplementary Fig. 9d–f), correlating with reduced glycolysis and PFKL expression in these cells (Supplementary Fig. 2).

Knocking down PFKL strongly impeded cell proliferation, to an extent comparable to that caused by TAp73 knockdown (Fig. 6b), indicating that PFKL also plays a critical role in the proliferation of tumor cells. To investigate the role of PFKL in TAp73-mediated cell proliferation, we over-expressed PFKL in *TAp73*^*−/−*^ MEFs and TAp73-knockdown U2OS cells, and the respective control cells. Overexpression of PFKL had minimal effect in control cells; however, it significantly restored the proliferation of MEFs and U2OS cells devoid of TAp73 (Fig. 6c, d). To examine the role of PFKL in TAp73-mediated oncogenic growth, we evaluated anchorage-independent growth in soft agar medium. The ability of *TAp73*^*−/−*^ MEFs to form colonies in soft agar was greatly reduced, to an extent that was only ~25% of that of *TAp73*^*+/+*^ MEFs (Fig. 6e). Interestingly, overexpression of PFKL in *TAp73*^*−/−*^ MEFs partially restored the anchorage-independent growth (Fig. 6e). To investigate the role of PFKL in the growth of tumor cells in animals, we injected *TAp73*^*+/+*^ MEFs expressing control vector, and *TAp73*^*−/−*^ MEFs expressing vector control or PFKL, to immune-compromised mice. As shown in Fig. 6f, expression of PFKL also significantly restored the ability of *TAp73*^*−/−*^ MEFs to produce tumors.

### Role of PFKL and G6PD in TAp73-mediated cell proliferation

**Fig. 7:**
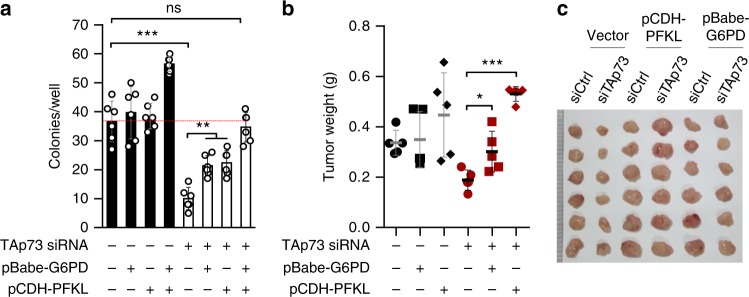
PFKL and G6PD mediate the tumorigenic effect of TAp73. **a** HCT116 cells stably expressing vector control, PFKL, G6PD, or both PFKL and G6PD were treated with control or TAp73 siRNA for 24 h, and 1000 cells for each condition were plated in soft agar for colony formation. Numbers of colonies with a diameter greater than 10 μm were quantified 10 d later (means ± S.D., *n* = 6). **b**, **c** HCT116 cells stably expressing vector, PFKL, or G6PD were treated with a control or TAp73 siRNA. Cells were xenografted into immunodeficient mice. Average weights (**b**, means ± S.D. indicated) and images (**c**) of xenograft tumors at 3 weeks are shown

To investigate the relative contributions of PFKL and G6PD in TAp73-mediated proliferation, we generated HCT116 cell line stably expressing exogenous PFKL and/or G6PD, and then knocked down TAp73 in these cells (Supplementary Fig. 10a). While cells overexpressing PFKL or G6PD alone showed no clear proliferative advantage in soft agar over the control cells, cells overexpressing both grew noticeably better (Fig. 7a and Supplementary Fig. 10b). As expected, depleting TAp73 resulted in a strong reduction in anchorage-independent growth in control cells, but only a moderate reduction in PFKL or G6PD overexpression cells (Fig. 7a and Supplementary Fig. 10b). In cells expressing both PFKL or G6PD, depleting TAp73 still reduced proliferation compared to TAp73-proficient cells; however, the TAp73-depleted cells grew as well as TAp73-proficient cells without PFKL/G6PD overexpression (Fig. 7a and Supplementary Fig. 10a). Thus, both PFKL and G6PD contribute the proliferative effect of TAp73, and their simultaneous activation by TAp73 likely affords tumor cells a strong advantage during anchorage-independent growth.

To investigate the role of PFKL and G6PD in tumor growth in vivo, we injected control, PFKL-overexpressing, and G6PD-overexpressing HCT116 cells, each with and without TAp73-knockdown, into immune compromised mice. Of note, in control cells, knocking down G6PD led to strong reduction in tumor growth (Fig. 7b, c). However, in PFKL- or G6PD-overexpressing cells, knocking down TA73 had a minimal effect on tumor growth (Fig. 7b, c). Moreover, unlike G6PD, expression of PFKL alone in control HCT116 cells led to a stronger tumorigenicity (Fig. 7b, c). Collectively, these findings demonstrate that upregulation of PFKL is strongly tumorigenic and is likely a major mechanism underlying the effect of TAp73 in tumor cells.

## Discussion

The Warburg effect is the most prominent metabolic feature associated with malignant transformation, presumably enabling a large-scale biosynthetic program required for active cell proliferation^1–4^. Here we show that TAp73, the transactivation-component isoform of p73, promotes the Warburg effect by stimulating the expression of PFKL. As the “gate-keeper” of glycolysis, PFK-1 is subjected to various modes of feed forward stimulation and feedback inhibition (Supplementary Fig. 1a)^5,11^. Notable among them is the inhibition by high levels of ATP. As it was first demonstrated in 1970s, glucose metabolism in proliferating cells is likely limited by ATP consumption, rather than ATP production^33^. Thus, relieving the inhibition of ATP on PFK-1 is an important mean by which tumor cells achieve the Warburg effect^34^. For example, the oncoprotein Akt increases PFK-2 to generate F-2,6-BP, a potent activator for PFK-1 that renders it less sensitive to ATP inhibition^14^. Tumor cells, as well as normal cells, can also increase the expression of PFK-1, although mechanisms are largely undefined^14^. The current study reveals TAp73 as a critical regulator of PFK-1 and, to our knowledge, the first transcriptional factor that specifically activates the L isozyme. Also importantly, TAp73 levels are highly responsive to growth conditions, engendering similar changes in PFKL levels (Fig. 3c–e). Thus, the TAp73-PFKL axis may represent an important way by which growth signals are coupled with glucose metabolism in proliferating normal and malignant cells. Moreover, the frequent up-regulation of TAp73 likely contributes to increased basal activity of PFK-1 and preferential up-regulation of PFKL over the other isoforms in tumor cells.

Although the rationale for the preferential use of glycolysis over the energetically much more efficient oxidative phosphorylation in proliferating cells remains unclear, it has been proposed that an enhanced glycolytic flux permits the diversion of glycolytic intermediates into subsidiary pathways for macromolecule synthesis^10,35,36^. The PPP, which branches off glycolysis at glucose-6-phosphate (G6P), provides cells with ribose for the synthesis of DNAs and RNAs, and the reducing equivalent NADPH for reductive biosynthesis and ROS detoxification^24^. In tumor cells, the PPP flux is often up-regulated in addition to a higher glycolytic flux^24,37^. We previously found that TAp73 activates G6PD^25^, while the current study shows that TAp73 stimulates the expression of 6PGD (Fig. 2j and Supplementary Fig. 3a), another key PPP enzyme for NADPH production. Thus, TAp73 acts as a prominent regulator of the PPP.

Nevertheless, a hyperactive PPP is expected to consume more G6P, raising an important question as to how proliferating cells maintain glycolysis in the face of reduced levels of the shared starting metabolite. The activation of PFKL by TAp73 likely provides an explanation, and underscores a coordinated regulation of glycolysis and the PPP. This coordination is critical for oncogenic growth, as overexpression of PFKL and G6PD simultaneously, but not separately, near-completely restores the growth defects of TAp73-deficient cells under both anchorage-dependent and anchorage-independent conditions. Moreover, upon G6PD or PFKL overexpression in HCT116 cells, knockdown of TAp73 no longer impairs tumor growth in xenograft mouse models. Overexpression of PFKL alone also enhances tumorigenic potential of TAp73-proficient cells. Thus, both G6PD and PFKL appear to have an profound effect on tumorigenesis.

Although the TAp73 are highly similar to p53 and p63 in its DNA-binding domain, TAp73—but not p53 or p63—is capable of inducing PFKL expression. p73 (and p63) is considered to be an ancestral member of the p53 family^38,39^. Unlike p53 whose deficiency resulted in no major developmental defects, deficiency in TAp73 leads to death of a significant fraction of newborn mice and severe development defects, especially in the central nervous system, in the remaining ones^20^. As glucose is the only fuel that the brain uses under non-starvation conditions, the defective glucose metabolism due to reduced PFKL and G6PD activities likely contributes to the developmental defects caused by TAp73 deficiency. p53 can suppress the PPP through a direct inactivation of G6PD^37^, and inhibit glycolysis by inducing the expression of TIGAR (TP53-inducible glycolysis and apoptosis regulator), a fructose-2,6-bisphosphatase that hydrolyzes the PFK-1 activator F-2,6-BP^40^. The opposing effects of TAp73 and p53 on both glycolysis and the PPP are remarkable, suggesting that the regulation of glucose metabolism might be a primordial activity of this important protein family. p73 and p53, as well as p63, regulate additional metabolic pathways^41,42^. Moreover, the intricate connection between the evolutionarily ancient p53 family and metabolic enzymes is consistent with the notion that the mutations in oncogenes and tumor suppressors are clonally selected during tumorigenesis, at least in part, due to their benefit in conferring metabolic adaptation^4,43^.

In summary, our findings identify a previously unanticipated mode of control for the committed step in glycolysis, define a critical role of TAp73 in the Warburg effect, and, along with our previous studies^25^, reveal a mechanism by which the two major glucose metabolic pathways, glycolysis and the PPP, are coordinated to support cell proliferation. They also underline metabolism as a main effector mechanism for the p53 family proteins in regulating cell proliferation and tumorigenesis. Given that PFK-1, especially PFKL, has a strong effect on glycolysis and may serve as a critical regulatory point during oncogenic transformation, reducing the activity of PFKL may provide therapeutic benefits.

## Methods

### Antibodies and reagents

Antibodies against the following proteins/epitopes were used for immunoblot with the sources, catalog numbers, and dilutions indicated: Actin (Sigma-Aldrich, St Louis, MO; A2066, 1:5000), Flag (Sigma-Aldrich, F3165, 1:10,000), p73 (Bethyl Laboratories, Montgomery, TX; A300–126A, 1:1000), G6PD (Sigma-Aldrich, HPA000834, 1:1000), p21 (BD Bioscience, San Jose, CA; 556431, 1:1000), PFKL (Santa Cruz Biotechnology, Dallas, TX; sc-292523, 1:1000) (Abcam, Cambridge, UK; ab181064, 1:2000), p53 (Santa Cruz sc-126HRP, 1:2000), PFKM (R&D Systems, Minneapolis, MN; MAB7687, 1:1000), PFKP (Cell Signaling Technology, Danvers, MA; 8164S, 1:1000), 6PGD (Abgent, San Diego, CA; AP5448c, 1:1000) (Santa Cruz, sc-39877, 1:500), and V5 (EASYBIO, BE2033, 1:2000).

Etoposide (ETO) was purchased from Selleck. The following reagents were purchased from Sigma-Aldrich: ATP, AMP, NAD^+^, NADH, NADP^+^, NADPH, doxorubicin (DOX), crystal violet (CV), 2′,7′-Dichlorofluorescin diacetate (DCF), citrate, fructose 6-phosphate (F6P), triose phosphate isomerase (TPI), aldolase (ALDO), and α-glycerophosphate dehydrogenase (GAPDH).

### Cell culture

Cells were maintained in standard culture medium without any antibiotic. *TAp73*^*−/−*^, *ΔNp73*^*−/−*^, and the corresponding wild-type MEFs have been previously described^20,26^. Genotypes were confirmed by PCR analysis^20^. Sense and antisense primers used for the wild-type *Trp73* were 5′-CTGGTCCAGGAGGTGAGACTGAGGC-3′ and 5′-CTGGCCCTCTCAGCTTGTGCCACTTC-3′, respectively. Sense and antisense primers for *TAp73*^*-/-*^ allele were 5′-GTGGGGGTGGGATTAGATAAATGCCTG-3′ and 5′-CTGGCCCTCTCAGCTTGTGCCACTTC-3′, respectively. Predicted PCR product sizes were 1.0 and 1.2 kb for the wild-type Trp73 and *TAp73*^*−/−*^ alleles, respectively.

HeLa, 293T, HCT116, A549, A172 and H1299 cells were from ATCC (Manassas, VA). SF188 cells were kindly provided by Dr. Craig. B Thompson (Memorial Sloan Kettering Cancer Center, New York, USA). All cells were cultured in a 5% CO_2_ humidified incubator (ThermoFisher Scientific, USA) at 37 °C. 293T, HCT116, HeLa, A549, A172, and MEF cell lines were maintained in standard Dulbecco’s modified Eagle’s medium (DMEM) (ThermoFisher Scientific, C11995500BT) with 10% fetal bovine serum (FBS) (GEMINI, 100–106). U2OS cells were cultured in McCoy’s 5 A Medium, and SF188 cells in DMEM supplemented with 2 mM additional l-glutamine. H1299 cells were cultured in standard RPMI-1640 medium (ThermoFisher Scientific, 11875093) with 10% FBS, unless indicated otherwise. All cells were cultured without the addition of penicillin-streptomycin and for no more than 2 consecutive months, and were routinely examined for mycoplasma contamination. Additionally, short tandem repeat (STR) profiling method were used to authenticate cell lines as described previously^44^.

### shRNA and siRNA

Expression plasmids for shRNAs were made in a pLKO.1-puro vector. The sequences were: p73 #1, 5′-ATCCGCGTGGAAGGCAATAAT-3′ (sense) and 5′-ATTATTGCCTTCCACGCGGAT-3′ (antisense); p73 #2, 5′-CTGTCATGGCCCAGTTCAATC-3′ (sense) and 5′-GATTGAACTGGGCCATGACAG-3′ (antisense); p73 #3, 5′-CCAAGGGTTACAGAGCATTTA-3′ (sense) and 5′-TAAATGCTCTGTAACCCTTGG-3′ (antisense); PFKL #1, 5′-GCTCCATCGATAACGACTTCT-3′ (sense) and 5′-AGAAGTCGTTATCGATGGAGCT-3′ (antisense); PFKL #2, 5′-CCTAGTGGGCTCCATCGATAA-3′ (sense) and 5′-TTATCGATGGAGCCCACTAGGT-3′ (antisense); PFKL #3, 5′-CTGAAGATGCTGGCACAATAC-3′ (sense) and 5′-GTATTGTGCCAGCATCTTCAGT-3′ (antisense). The following siRNAs were purchased from Life Technologies: p73, 5′-GAGCUCGGGAGGGACUUCAACGAAG-3′; TAp73, 5′- CGGAUUCCAGCAUGGACGU-3′; PFKL, 5′-GCACAAUACCGCAUCAGUATT-3′; p53, 5′-CCGCCUGAGGUUGGCUCUGACUGUA-3′; G6PD, 5′-ACGAGCUGAUGAAGAGAGUGGGUUU-3′, HK2, 5′- CCUGGGUGAGAUUGUCCGUAA-3′, GLUT1 5′-CGAACUAUGAACUACAAAGCUUCUA-3′. siRNAs were transfected into cells using Lipofectamine RNAiMAX transfection Agent (Invitrogen, Carlsbad, CA) following the manufacturer’s instruction. Stable shRNA transfectants were selected in medium containing 1 µg/ml puromycin (Calbiochem, San Diego, CA, catalog No: 540222) as previously described^45^.

### Semi-quantitative RT–PCR and quantitative RT–PCR

Total RNA was isolated from cells by Trizol and 2 µg RNA of each sample was reversed transcribed to cDNA by First-strand cDNA Synthesis System (Thermo scientific, catalog No. K1622). 0.2 µg cDNA of each sample was used as a template to perform PCR/quantitative PCR. Quantitative PCR were performed on CFX96 Real-Time PCR System (Bio-Rad, USA) and the amplifications were done using the SYBR Green PCR Master Mix (Gene star, China). The primer pairs for human genes were: *G6PD*, 5′-AGGCTGCAGTTCCATGATGT-3′ and 5′-ATCTGTTGCCGTAGGTCAGG-3′; *β-actin*, 5′-GACCTGACTGACTACCTCATGAAGAT-3′ and 5′-GTCACACTTCATGATGGAGTTGAAGG-3′; *p53*, 5′-CACGAGCTGCCCCCAGG-3′ and 5′-TCAGTCGACGTCTGAGT-3′; *TAp73*, 5′-GCACCTACTTCGACCTTCCC-3′ and 5′-GTAGTCATGCCCTCCAGGTG-3′. *PFKL*, 5′-GTGGTTGTCGGAGAAGCTGCGC-3′ and 5′-CGGTGCTCGAAATCAGTGTCT-3′; *PFKM*, 5′-TGAGGAGGCTACGAAGTCCA-3′ and 5′-TCTGGGCAGTGGTAGTGATG-3′; *PFKP*, 5′-CGCCTACCTCAACGTGGTG-3′ and 5′-ACCTCCAGAACGAAGGTCCTC-3′; *p21*, 5′-CCGGCGAGGCCGGGATGAG-3′ and 5′- CTTCCTCTTGGAGAAGATC-3′; *HK2* 5′-CCTGAGGACATCATGCGAGG-3′ and 5′-TGGACTTGAATCCCTTGGTCC-3′; *GLUT1* 5′-CAGCAAGAAGCTGACGGGT-3′ and 5′-CAGGATGCTCTCCCCATAGC-3′. Primers for mouse genes were: *G6PD*, 5′- GCCACTCCAGAAGAAAGACCT-3′ and 5′-GGCAAGGCCAGGTAGAATAG-3′; *β-actin*, 5′-ACTACATTCAATTCCATC-3′ and 5′-CTAGAAGCACTTGCGGTG-3′; *PFKL*, 5′-TTGTGATCGCATCAAGCAGT-3′ and 5′-GGATGTTGAAAGGGTCCTCA-3′; *PFKM*, 5′-TGGCACAGTGATTGGAAGTG-3′ and 5′-GCTCCACTCTGAACGGAAAG-3′. *PFKP*, 5′-GGGACCATCATCGGTAGTGC-3′ and 5′-GTCCGCTCCACTCCTTTCG-3′.

### Constructs

The coding sequences corresponding to the full-length human *TAp73* and *PFKL* genes were amplified by polymerase chain reaction (PCR) from cDNA library of 293T cells and then cloned into PCDH-puro-v5 empty vector as indicated. The cloning sequences are as follows: Human *TAp73*, 5′-CGCGGATCCATGGCCCAGTCCACCG-3′ (forward), and 5′-ATAAGAATGCGGCCGCTCAGTGGATCTCGGCCTCC-3′ (reverse). Human *PFKL*, 5′-CTAGCTAGCATGGGAGACTACAAGGACGATGATG-3′ (forward), and 5′-ATTTGCGGCCGCTCAGAAGCCCTTGTCCATGC-3′ (reverse). All constructs were confirmed by DNA sequencing.

### Chromatin immunoprecipitation (ChIP) and reporter assays

To identify potential p53 family protein response elements, we scanned the PFKL gene using the Genomatix Promoter Inspector Program (Genomatix Inc, Munich, Germany, software, http://www.genomatix.de). For ChIP assays, cells were cross-linked with 1% formaldehyde for 15 min at room temperature. Cross-linking was stopped by the addition of 100 mM Tris-HCl, pH 9.4. Cell lysates were sonicated to generate DNA fragments with an average size below 1000 bp and immunoprecipitated with indicated antibodies. Bound DNA fragments were eluted and amplified by PCR. Primer pairs were: RE1, 5′-CGCCTCGAGTTCCCCTCTCAGAGTGGGACTC-3′ and 5′-CGCAAGCTTGTACAGAGGCCGCAGGGCCTAG-3′; RE2, 5′-CGCCTCGAGACCCTCCACTCTACTTTCTGT-3′ and 5′-CGCAAGCTTGCCAAAAGGTGGAAGCACCCAG-3′; RE3, 5′-CGCCTCGAGCTGCCAGTGTTGCCCAGTCC-3′ and 5′-CGCAAGCTTGGCCTGTTTCAAGTCTTCTAG-3′.

For reporter assay, the PFKL genomic fragment (−3962 to −3712) containing RE1 (GCCCCAGCCCCATCATGCCC), fragment (−1725 to −1475) containing RE2 (ATCCATGTTGTAGCATGTGC), and fragment (+2599 to +2849) containing either the wild-type (CCACAAGTCCTGTCCTGTGT) or mutant (CCATAATTCCTGTTCTTTGT, with mutated nucleotides underlined) p73-binding region (RE3) were cloned into pGL3-basic vector (Promega, Madison, WI, USA, catalog No: E1751). Luciferase reporter assays were performed as described previously^25,42^. Briefly, the reporter plasmids were transfected into 293T cells together with a Renilla luciferase plasmid and increasing amounts of plasmids expressing TAp73 protein. Twenty-four hours after transfection, the luciferase activity was determined using a dual Luciferase Assay System (Promega, catalog No: E1910). Transfection efficiency was normalized on the basis of the Renilla luciferase activity.

### Measurements of NADH, NADPH, and ROS levels

NADH and NADPH levels were determined using the NAD^+^/NADH Quantification kit (BioVision, Mountain View, CA, USA, catalog No: K337) and NADP^+^/NADPH Quantification kit (BioVision, Mountain View, CA, USA, catalog No: K347), respectively. ROS levels were analyzed as described^46^. Briefly, cells were incubated at 37 °C for 30 min in medium containing 10 µM 2′,7′-dichlorodihydrofluorescein diacetate (DCF). Cells were then washed twice with PBS, trypsinized and re-suspended in PBS. Fluorescence was immediately measured using a FACScan Flow Cytometer (Becton Dickinson, San Jose, CA).

### Cell proliferation assay and crystal violet (CV) staining of cells

Cell proliferation assay were performed as described^42^. Briefly, cells were transfected with siRNAs for 24 h and seeded in 6-well cell culture dishes in triplicates at a density of 5000 or 20,000 cells per well in 2 ml of medium supplemented with 10% FBS. The medium was changed every other day. Cell number at the indicated time points was determined by counting using a hemocytometer. For CV staining, cells were fixed with 10% formalin for 5 min and stained with 0.05% CV for 30 min. After washed with distilled water, cells were photographed.

### Extracellular acidification rate (ECAR) and oxygen consumption rate (OCR)

ECAR and OCR were analyzed on a XF96 Extracellular Flux Analyzer (Seahorse Bioscience) as previously described^47,48^. Cells were plated in non-buffered DMEM media with 10 mM glucose. Measurements were obtained under basal conditions and after the addition of 2 μM oligomycin and 100 mM 2-DG.

### Western blotting

Whole-cell lysates were made in modified RIPA lysis buffer (10 mM Tris-HCl at pH 7.5, 5 mM EDTA, 150 mM NaCl, 1% NP-40, 1% Sodium deoxycholate, 0.025% SDS, and complete protease cocktail) for 15 min on ice, and boiled in 2x loading buffer. Protein samples were resolved by SDS-PAGE and transferred onto nitrocellulose membrane, which was blocked in 5% skim milk in TBST and probed with the indicated antibodies. Uncropped scans of the blots are provided in the Supplementary Figs. 11–13.

### Glycolytic flux measurements

The flux of glycolysis was measured based on the rate of glucose consumption and the ratio of ^13^C incorporated into lactate determined by LC-MS. Briefly, cells were cultured in medium with or without [1,2–^13^C_2_]glucose. After 12 h, medium was collected and cells were treated with cold 80% methanol. Metabolites were extracted and analyzed by LC-MS. Flux analysis was performed on TSQ Quantiva Triple Quadrupole mass spectrometer (Thermo Fisher Scientific, San Jose, CA) with positive/negative ion switching. MRM mode was used for data acquisition. Mobile phase A was prepared by adding 2.376 ml tributylamine and 0.858 ml acetic acid to HPLC-grade water, then adding HPLC-grade water to 1 l volume. Mobile phase B was HPLC-grade methanol. Synergi Hydro-RP 100 A column was used for polar metabolites separation with column temperature at 35 °C. The measured mass isotopomer distributions were corrected by natural abundances.

### Xenograft tumor models

Xenograft study was performed as described^25^. Briefly, cells were injected subcutaneously into the flanks of 3-to 4-week-old athymic Balb-c nu/nu male mice. Tumor growth was evaluated at 2 or 3 weeks post-injection as indicated. All animal experiments were performed in accordance with relevant guidelines and regulations and were approved by the Animal Care and Use Committee at Tsinghua University.

### PFK-1 enzyme activity

PFK-1 enzyme activity was determined as described^49^. Briefly, fresh cell lysates were added into to a reaction mixture containing 50 mM Tris-HCl pH 7.5, 100 mM KCl, 5 mM MgCl_2_, 1 mM ATP, 0.2 mM NADH, 5 mM Na_2_HPO_4_, 0.1 mM AMP, 1 mM NH_4_Cl, 5 mM fructose-6-phosphate, 5 units triose phosphate isomerase, 1 unit aldolase., and 1 unit α-glycerophosphate dehydrogenase. The decrease in absorbance at 340 nm as a result of NADH oxidation was measured every 10 s for 15 min on a SpectraMax® M2e Microplate Reader (Molecular Devices, CA, USA).

### Human colon cancer samples assessment

Human colon cancer tissues and their adjacent tissues were obtained with the patients informed consent from Peking Union Medical College Hospital (Beijing, China). All the procedures were performed under the permission of the Peking Union Medical College Hospital Ethics Board. Tissue samples were analyzed by quantitative RT-PCR. Total RNA was isolated from ~30 mg of each tissue sample using Trizol reagent (Invitrogen), following the manufacturer’s instructions. GAPDH was used as internal quality control. The primer pairs for human *GAPDH* gene are: 5′-GGAGCGAGATCCCTCCAAAAT-3′ and 5′-GGCTGTTGTCATACTTCTCATGG-3′.

### Statistical analysis

A two-tailed Student’s *t*-test was performed and expressed as a *P* value. GraphPad Prism 7 (for windows) and Excel were used to perform statistical analysis. Animal experiments were randomized, and no exclusion were performed from the experiments.

## Electronic supplementary material

Supplementary Information

Description of Additional Supplementary Files

Supplementary Data 1

Supplementary Data 2

## Data Availability

All relevant data are available from the corresponding authors upon reasonable request. The source data underlying Figs. 1a–i, 2a–c, f, g, j, 3a, c, 4b–d, k, 5a–i, 6a–c, e, f, 7a, b, and Supplementary Figs. 12b, 2d-k, 3a, 4a, 4b, 5a-d, 6b-g, 7a, 9b, 9d–g, and 10a are provided as Source Data files (Supplementary Data 1, 2).
