# Supplementary material for: TAp73-induced phosphofructokinase-1 transcription promotes the Warburg effect and enhances cell proliferation
Source: Nat Commun. 2018 Nov 8;9:4683. doi: 10.1038/s41467-018-07127-8 (PMC6224601; doi:10.1038/s41467-018-07127-8)
Supplement: Supplementary file 1 — Supplementary Information [file 41467_2018_7127_MOESM1_ESM.pdf]

Supplementary Information

TAp73-induced phosphofructokinase-1 transcription promotes the Warburg effect and cell proliferation

Li et al.

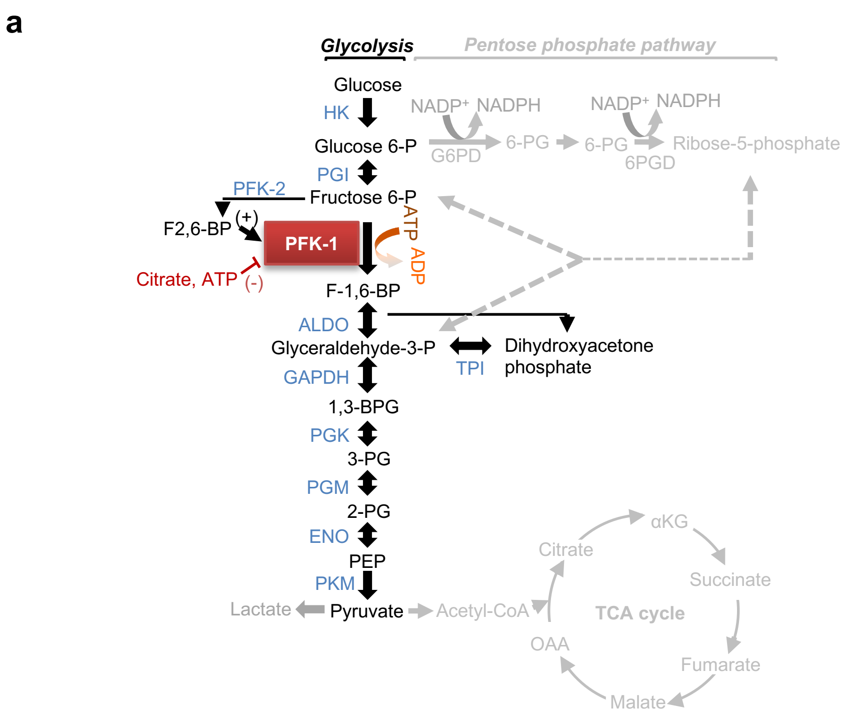

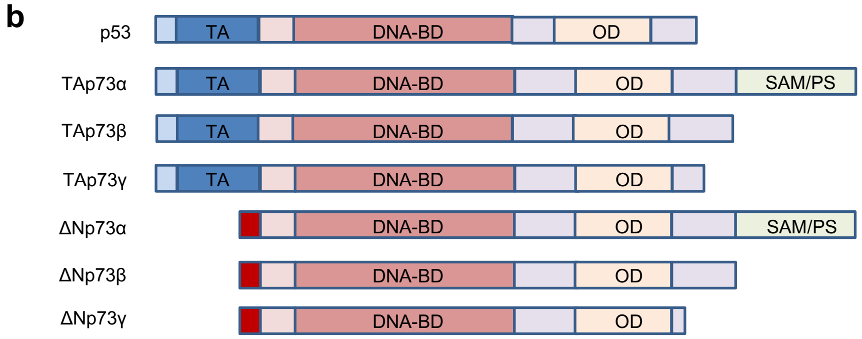

Supplementary Fig. 1 Glucose metabolic pathways and p73 isoforms. **a**, A schematic representation of glycolysis, the PPP and TCA cycle. Glucose 6-P, glucose 6-phosphate; Fructose 6-P, fructose 6-phosphate; F-1,6-BP, fructose 1,6-biphosphate; F-2,6-BP, fructose 2,6-biphosphate; Glyceraldehyde-3-P, glyceraldehyde-3-phosphate; 1, 3-BPG, 1,3-bisphosphoglycerate; 3-PG, 3-phosphoglycerate; 2-PG, 2-phosphoglycerate; 6-PG, 6-phosphogluconate; PEP, phosphoenolpyruvate; αKG, α-ketoglutarate; TCA cycle, tricarboxylic acid cycle; HK, hexokinase; PGI, phosphoglucose isomerase; PFK1, phosphofructokinase 1; PFK2, phosphofructokinase 2; ALDO, aldolase; TPI, triosephosphate isomerase; GAPDH, glyceraldehyde 3-phosphate dehydrogenase; PGK, phosphoglycerate kinase; PGM, phosphoglyceratemutase; ENO, enolase; PKM, pyruvate kinase; G6PD, glucose-6-phosphate dehydrogenase; 6PGD, 6-phosphogluconate dehydrogenase. **b**, Schematic representation of p73 isoforms and p53. Each p73 isoform comprises different splicing variants (α, β, γ, etc.). TA, transactivation domain; DBD, DNA-binding domain; OD, oligomerization domain; SA M, sterile α motif.

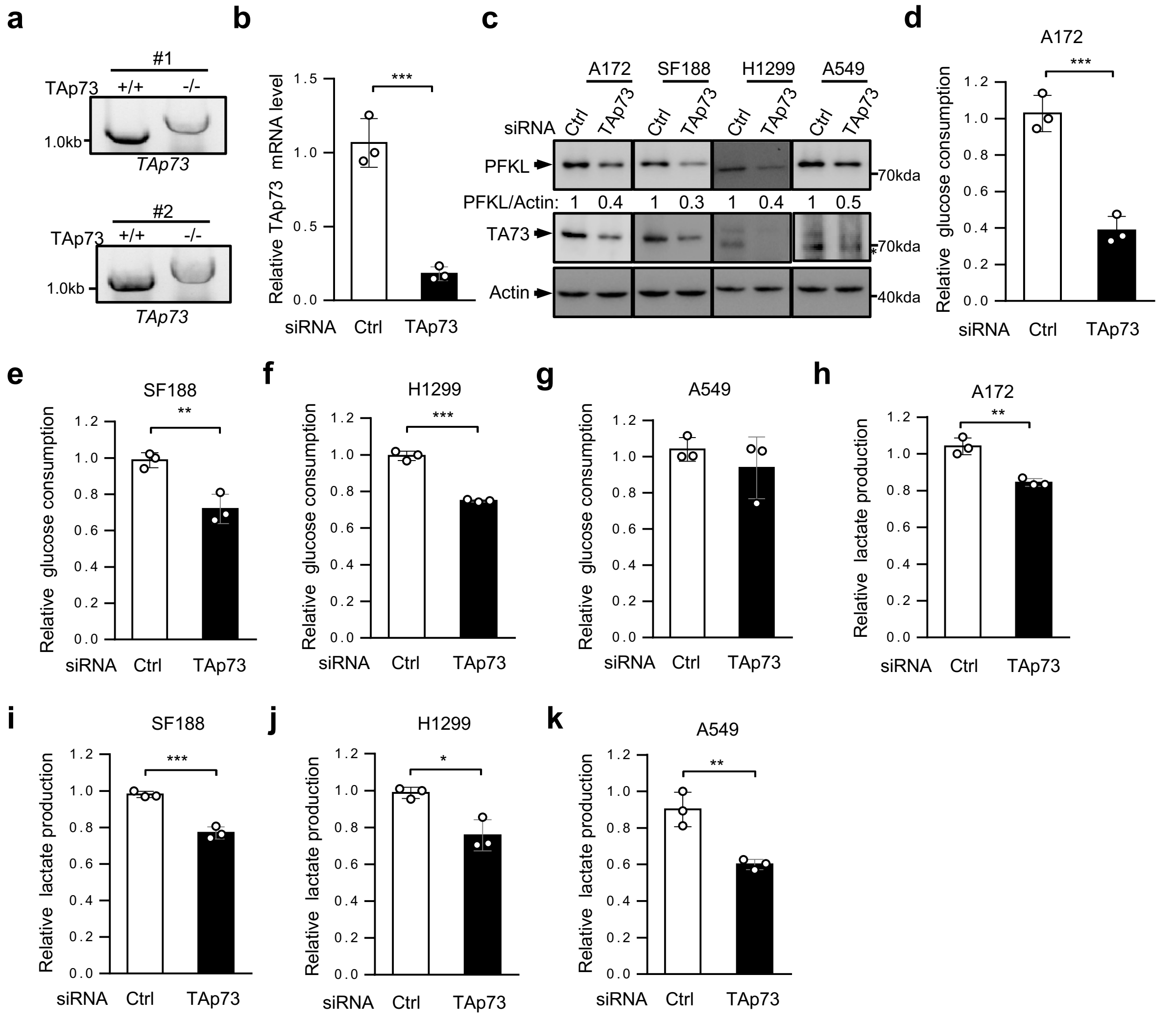

Supplementary Fig. 2 Knockdown of TAp73 decreases glycolysis and PFKL expression in multiple cell lines. **a**, Genotypes of two independent pairs (#1 and #2) of *TAp73*$^{+/+}$ and *TAp73*$^{-/-}$ MEFs were confirmed by PCR analysis as described previously[1]. Predicted PCR product sizes were 1.0 kb and 1.2 kb for the wild-type *Trp73* and *TAp73*$^{-/-}$ alleles, respectively. Related to Fig. 2a, b. **b**, U2OS cells treated with control (Ctrl) or *TAp73* siRNA were assayed TAp73 expression by qRT-PCR analysis (n = 3). Related to Fig. 2d.c, A172, SF188, H1299 and A549 cells were transfected with control or *TAp73* siRNA for 48 h. Protein expression was measured by Western blot analysis (**c**). Results are representative of three independent experiments. **d-k**, Relative glucose consumption (**d-g**, n=3) and lactate excretion (**h-k**, n=3) in A172 (**d**, **h**), SF188 (**e**, **i**), H1299 (**f**, **j**) and A549 (**g**, **k**) cells. Data are shown as means ± S.D., two-tailed unpaired Student's t-test, * $P < 0.05$, ** $P < 0.01$, and *** $P < 0.001$.

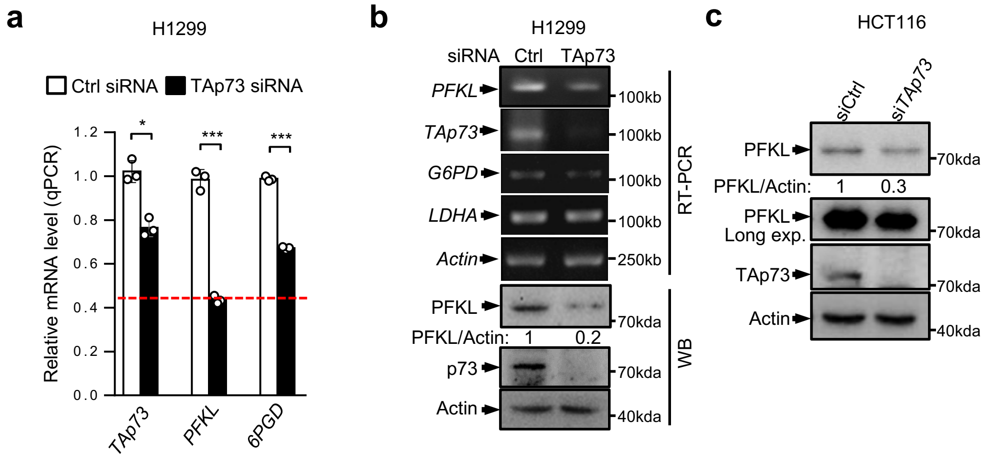

Supplementary Fig. 3 PFKL is a target for TAp73. **a**, **b**, H1299 cells were transfected with control or *TAp73* siRNA. PFKL expression was analyzed by qRT-PCR (**a**, n=3), semi-quantitative RT-PCR, and Western blot (**b**). Data are shown as means ± S.D., two-tailed unpaired Student's t-test, * $P < 0.05$ and *** $P < 0.001$. **c**, Western blot analysis of PFKL expression in HCT116 cells treated with control or *TAp73* siRNA as indicated. Results are representative of three independent experiments.

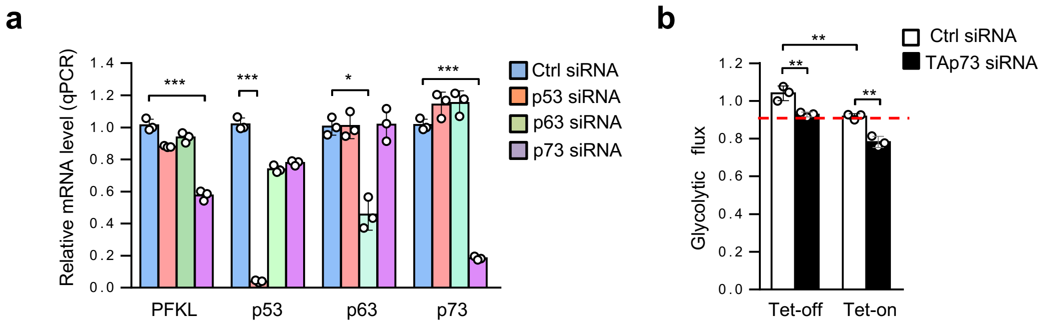

Supplementary Fig. 4 p73, not p53 and p63, regulates PFKL expression, and p53 minimally affects TAp73-mediated glycolysis. **a**, U2OS cells were transfected with control, *p53*, *p63*, or *p73* siRNA as indicated, and the indicated RNAs were analyzed by qRT-PCR (n=3). **b***,* $p53^{-/-}$ HCT116 cells stable expressing Tet-inducible p53 were cultured in medium containing [1,2-$^{13}C_2$]glucose and treated with doxycycline to induce p53 expression (Tet-on). Relative glycolytic flux is shown (n=3 biological independent wells). Data are shown as means ± S.D., two-tailed unpaired Student's t-test, * $P < 0.05$, ** $P < 0.01$, and *** $P < 0.001$.

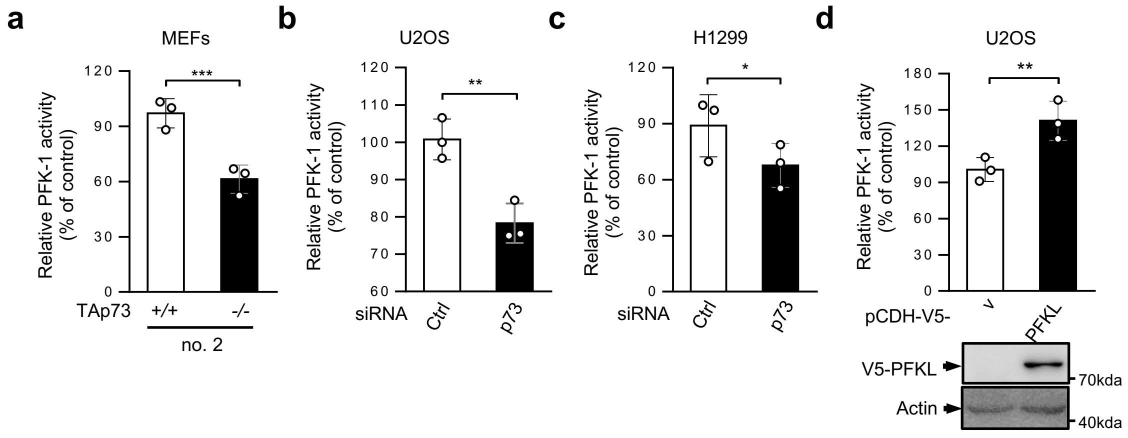

Supplementary Fig. 5 TAp73 positively regulates PFK-1 activity. **a-d,** Relative PFK-1 activity (n=3) in *TAp73*$^{+/+}$ and *TAp73*$^{-/-}$ MEFs (**a**), U2OS (**b**) and H1299 (**c**) cells treated with ether control or *p73* siRNA, and U2OS cells that stably expressing control vector or V5-PFKL (**d,** protein expression shown in the bottom). Data are shown as means ± S.D., two-tailed unpaired Student's t-test, * $P < 0.05$, ** $P < 0.01$, and *** $P < 0.001$.

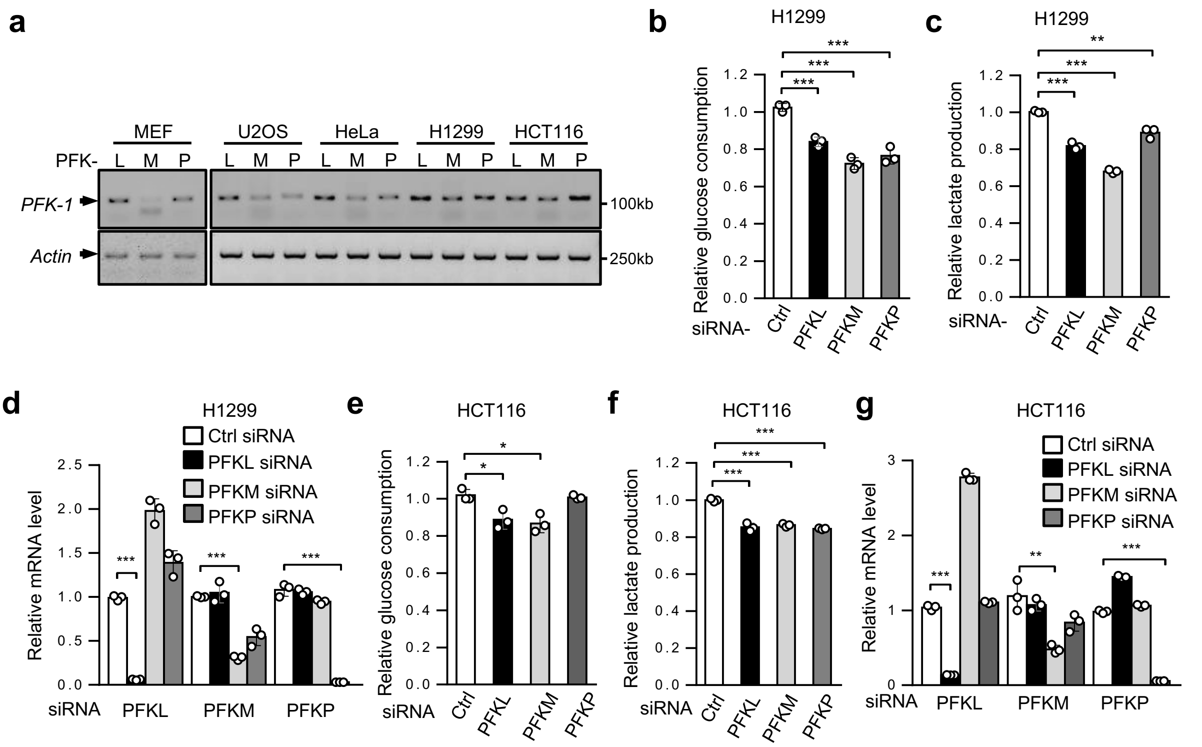

Supplementary Fig. 6 Effect of PFK-1 isoforms on glucose consumption and lactate production. **a**, mRNA levels of PFKL, PFKM and PFKP in different cell lines were analyzed by qRT-PCR. Results are representative of three independent experiments. **b-g**, H1299 (**b-d**) and HCT116 (**e-g**) cells were treated with control, PFKL, PFKM, or PFKP siRNA. Relative glucose consumption (**b**, **e**), lactate production (**c**, **f**), and mRNA levels (**d**, **g**) are shown. Data are means ± S.D. (n=3), two-tailed unpaired Student's t-test, * $P < 0.05$, ** $P < 0.01$, and *** $P < 0.001$.

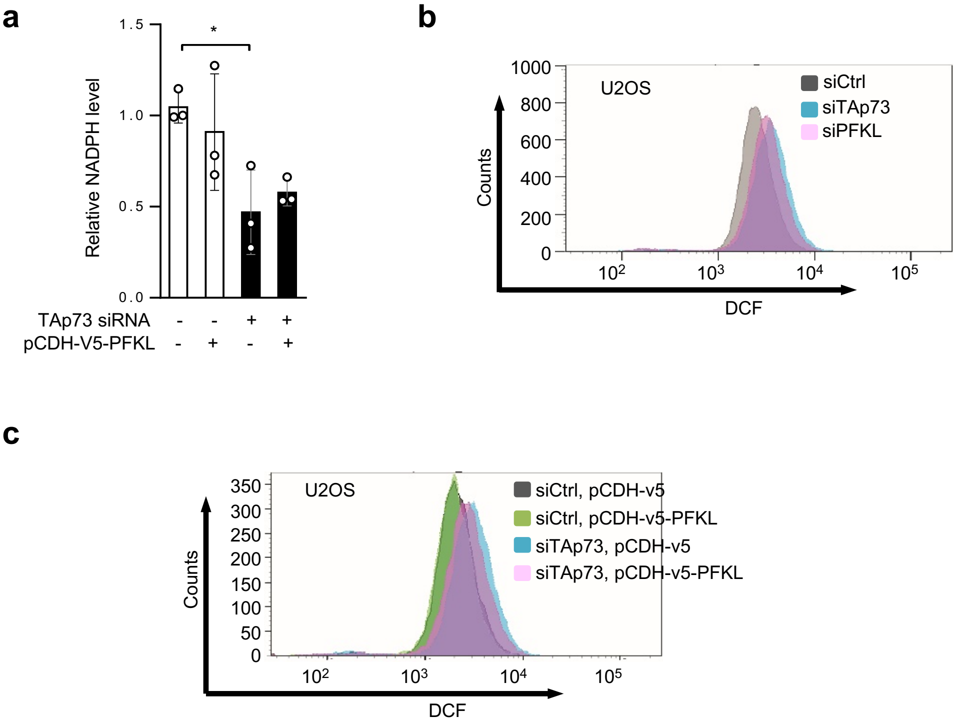

Supplementary Fig. 7 TAp73 suppresses ROS in part by activating PFKL. **a**, U2OS cells stably expressing control vector or PFKL were transfected with control or *TAp73* siRNA for 48 h. NADPH levels are shown. Data are means ± S.D. (n=3), two-tailed unpaired Student's t-test, * $P < 0.05$. **b**, U2OS cells treated with control, *TAp73*, or *PFKL* siRNA were assayed for ROS accumulation by 2'7'-dichlorodihydrofluorescein diacetate (DCF) staining and FACS analysis. Related to Fig. 5h. **c**, Representative images of ROS levels in U2OS cells stably expressing control or PFKL plasmid and treated with control or *TAp73* siRNA. Related to Fig. 5i.

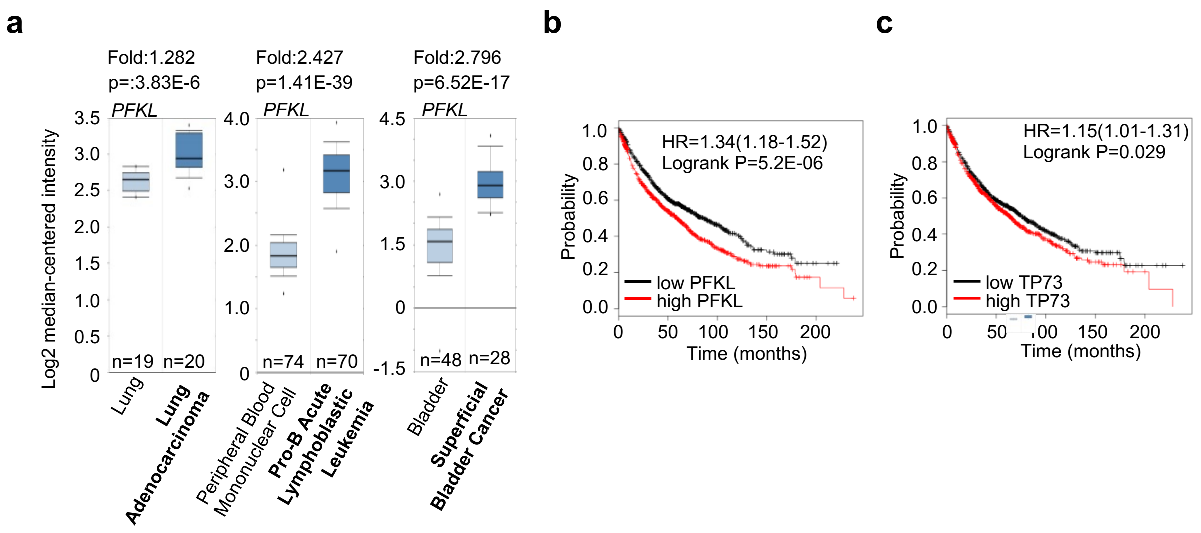

Supplementary Fig. 8 PFKL is overexpressed in human tumors and correlated to poor survival of human glioma patients. **a**, Box plot comparing *PFKL* transcript levels in lung adenocarcinoma, acute lymphoblastic leukemia, bladder cancer, and their normal counterparts. **b**, **c**, Kaplan–Meier survival curves of patients with glioma based on PFKL (**b**) or p73 (**c**) expression[10].

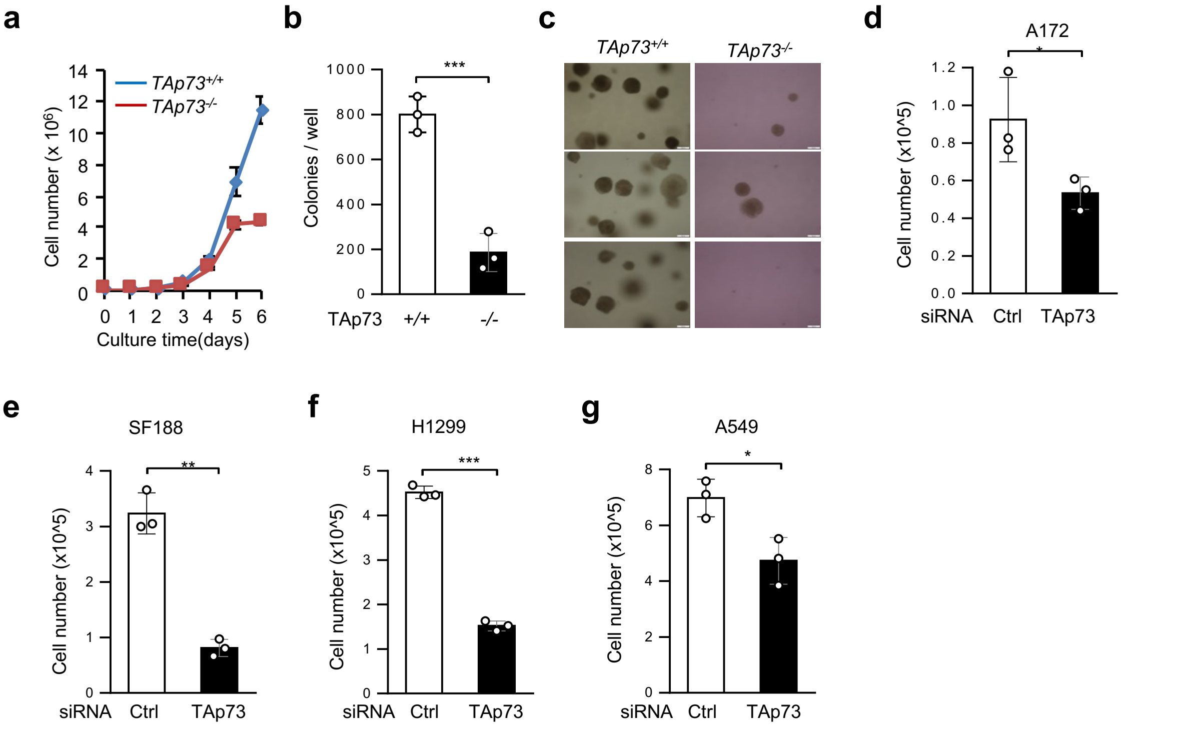

Supplementary Fig. 9 TAp73 promotes tumor cell proliferation through PFKL. **a**, Proliferation of *TAp73*$^{+/+}$ and *TAp73*$^{-/-}$ MEFs at day 6 (n=3). **b**, **c**, Colony formation assay of *TAp73*$^{+/+}$ and *TAp73*$^{-/-}$ MEFs. Shown are numbers of colonies with a diameter greater than 20 μm (b, n=3), and representative images of colonies stained with crystal violet at day 6 (**c**). **d-g**, Proliferation of A172 (**d**), SF188 (**e**), H1299 (**f**), and A549 (**g**) cells treated with control or *TAp73* siRNA as indicated (n=3). Protein expression is shown in Supplementary Fig. 2c. Data are means ± S.D., two-tailed unpaired Student's t-test, * $P < 0.05$, ** $P < 0.01$, and *** $P < 0.001$.

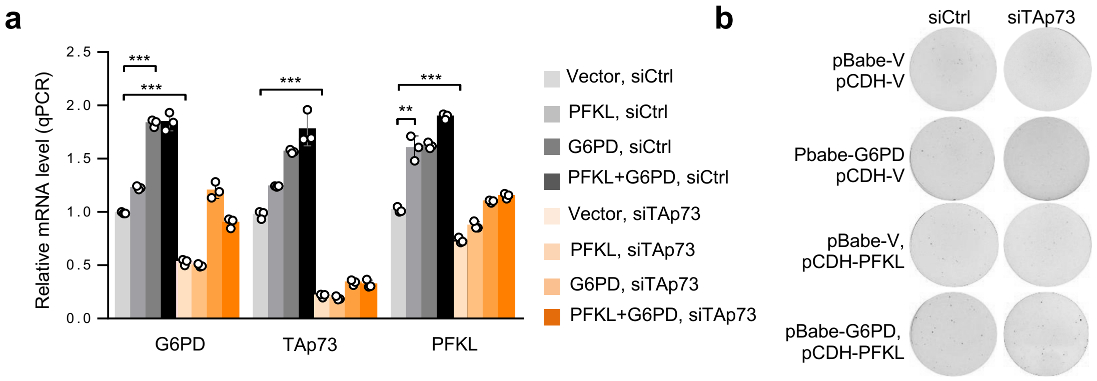

Supplementary Fig. 10 TAp73 promotes anchorage-independent growth of HCT116 cells through PFKL and G6PD (Related to Fig. 6a). HCT116 cells stably expressing vector control, G6PD, PFKL, or both G6PD and PFKL were transfected with *TAp73* siRNA or control siRNA. (**a**) mRNA levels of *PFKL*, *G6PD*, *TAp73*, and *actin* were analyzed by qRT-PCR (n=3). (**b**) Representative images of colonies stained with crystal violet at day 6. Data are means ± S.D., two-tailed unpaired Student's t-test, ** $P < 0.01$ and *** $P < 0.001$.

Supplementary Figure 11

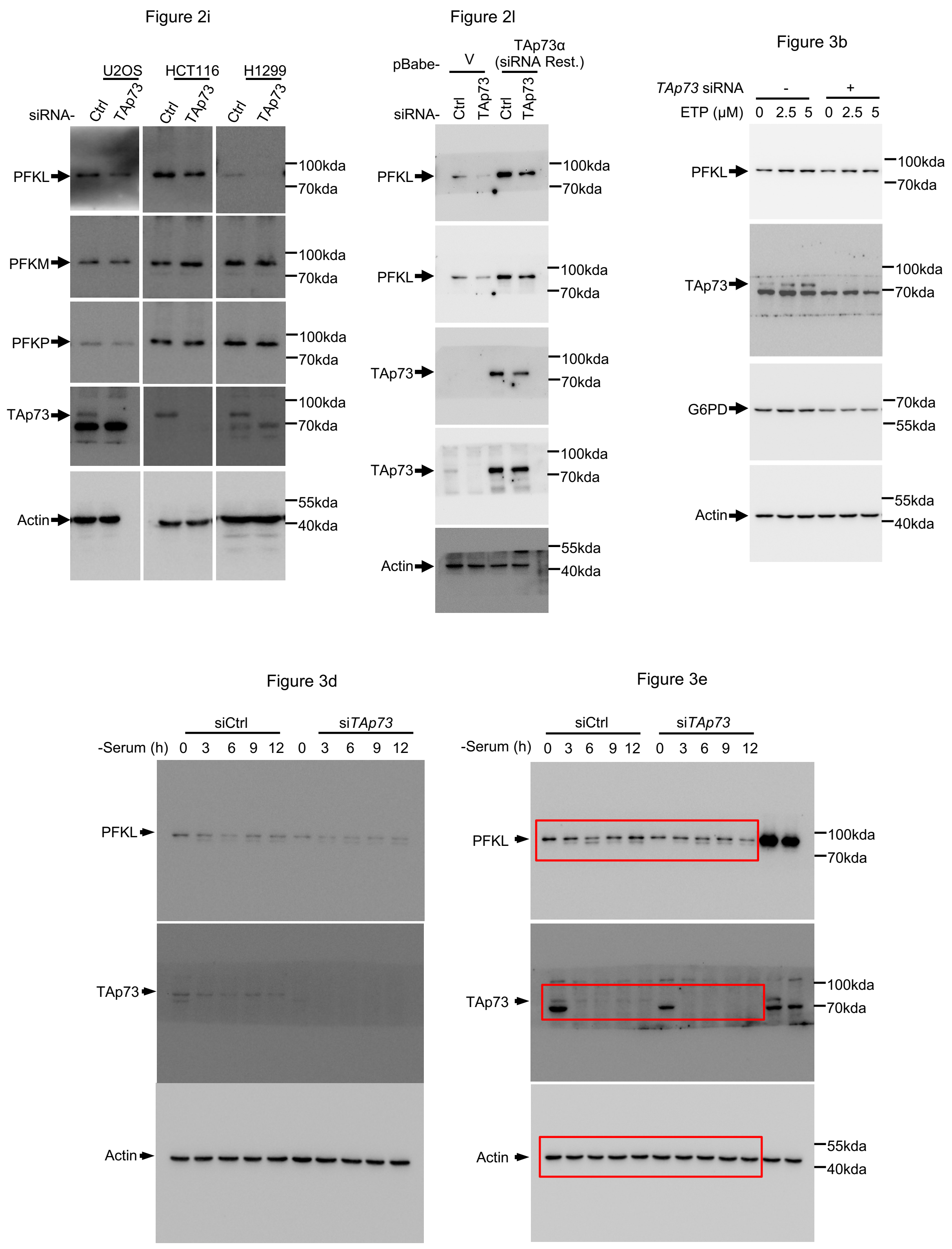

Supplementary Fig. 11 Uncropped western blot related to Figures 2 and 3.

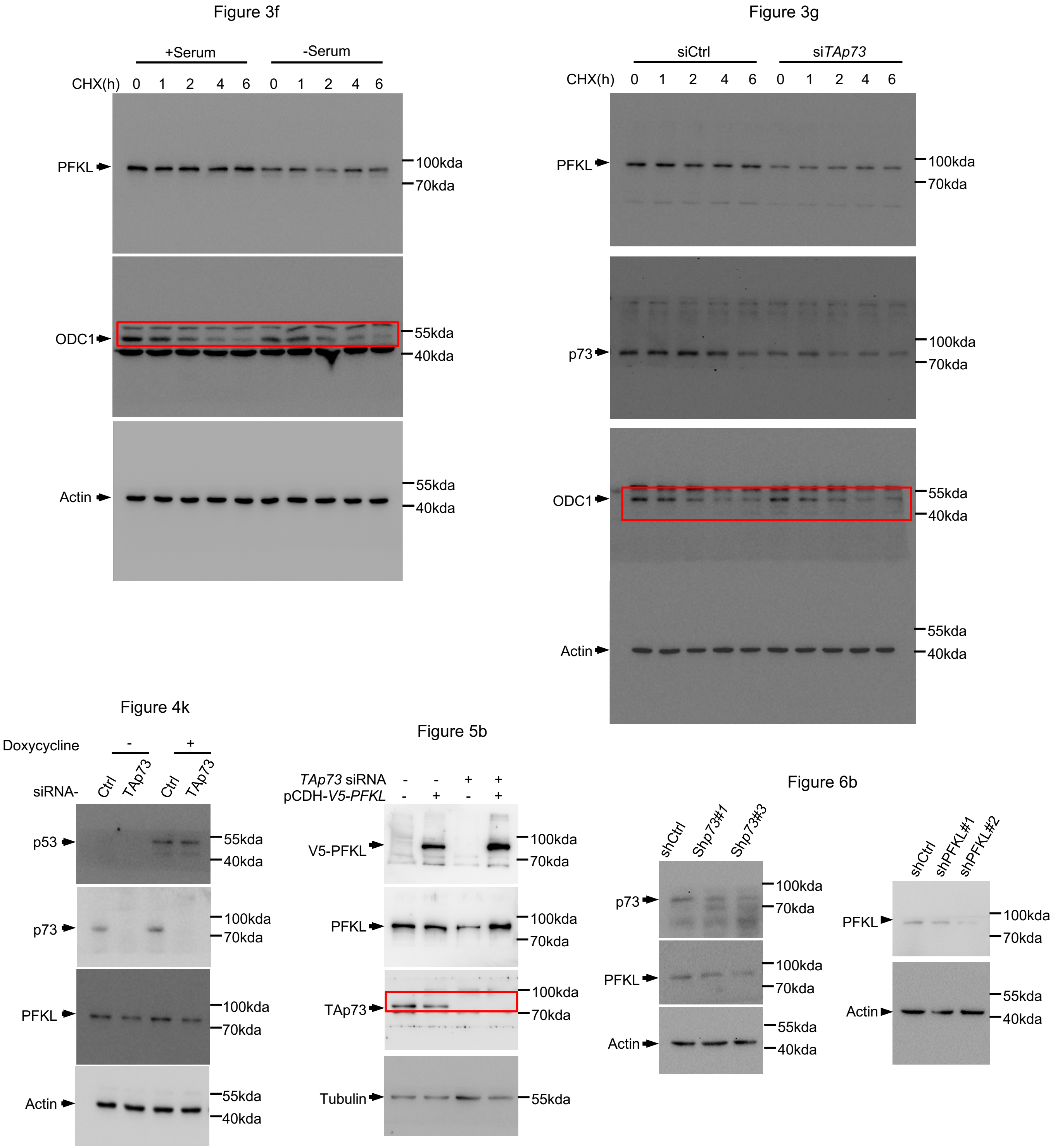

Supplementary Fig. 12 Uncropped western blot related to Figures 3-6.

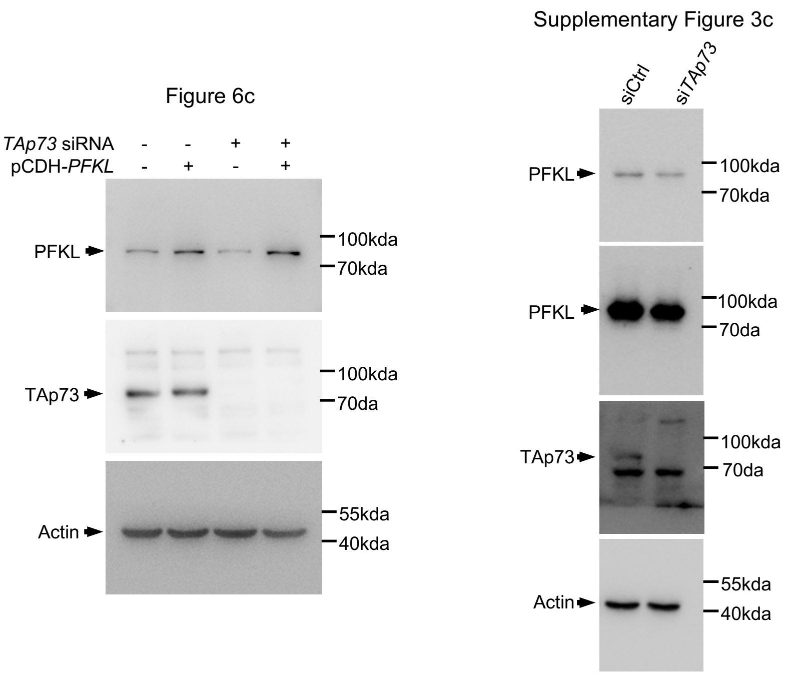

Supplementary Fig. 13 Uncropped western blot related to Figure 6 and Supplementary Figure 3.
