# Supplementary material for: TAp73-induced phosphofructokinase-1 transcription promotes the Warburg effect and enhances cell proliferation
Source: Nat Commun. 2018 Nov 8;9:4683. doi: 10.1038/s41467-018-07127-8 (PMC6224601; doi:10.1038/s41467-018-07127-8)
Supplement: Supplementary file 2 — Description of Additional Supplementary Files [file 41467_2018_7127_MOESM2_ESM.pdf]

Description of Additional Supplementary Files

File Name: Supplementary Data 1
Description: Source data for Figs 1a-i, 2a-c, 2f, 2g, 2j, 3a, 3c,4b-d, 4k, 5a-i, 6a-c, 6e, 6f, 7a and 7b.

File Name: Supplementary Data 2
Description: Source data for Supplementary Figs 12b, 2d-k, 3a, 4a, 4b, 5a-d, 6b-g, 7a, 9b, 9d-g, and 10a.
